# Graphene Oxide and Graphene Quantum Dots as Delivery Systems of Cationic Porphyrins: Photo-Antiproliferative Activity Evaluation towards T24 Human Bladder Cancer Cells

**DOI:** 10.3390/pharmaceutics13091512

**Published:** 2021-09-18

**Authors:** Luca Menilli, Ana R. Monteiro, Silvia Lazzarotto, Filipe M. P. Morais, Ana T. P. C. Gomes, Nuno M. M. Moura, Sara Fateixa, Maria A. F. Faustino, Maria G. P. M. S. Neves, Tito Trindade, Giorgia Miolo

**Affiliations:** 1 Department of Pharmaceutical and Pharmacological Sciences, University of Padova, 35131 Padova, Italy; luca.menilli@unipd.it (L.M.); silvia.lazzarotto.1@studenti.unipd.it (S.L.); 2 Department of Biology, University of Padova, 35131 Padova, Italy; 3 CICECO—Aveiro Institute of Materials, Department of Chemistry, University of Aveiro, 3810-193 Aveiro, Portugal; anarita.rvcm@ua.pt (A.R.M.); sarafateixa@ua.pt (S.F.); 4 LAQV—Requimte, Department of Chemistry, University of Aveiro, 3810-193 Aveiro, Portugal; filipemorais@live.ua.pt (F.M.P.M.); nmoura@ua.pt (N.M.M.M.); faustino@ua.pt (M.A.F.F.); 5 CESAM, Department of Biology, University of Aveiro, 3810-193 Aveiro, Portugal; ana.peixoto@ua.pt; 6 Center for Interdisciplinary Investigation (CIIS), Faculty of Dental Medicine, Universidade Católica Portuguesa, 3504-505 Viseu, Portugal

**Keywords:** bladder cancer, cationic porphyrins, graphene oxide, graphene quantum dots, non-covalent hybrids, photodynamic therapy

## Abstract

The development of new photodynamic therapy (PDT) agents designed for bladder cancer (BC) treatments is of utmost importance to prevent its recurrence and progression towards more invasive forms. Here, three different porphyrinic photosensitizers (PS) (TMPyP, Zn-TMPyP, and P1-C_5_) were non-covalently loaded onto graphene oxide (GO) or graphene quantum dots (GQDs) in a one-step process. The cytotoxic effects of the free PS and of the corresponding hybrids were compared upon blue (BL) and red-light (RL) exposure on T24 human BC cells. In addition, intracellular reactive oxygen species (ROS) and singlet oxygen generation were measured. TMPyP and Zn-TMPyP showed higher efficiency under BL (IC_50_: 0.42 and 0.22 μm, respectively), while P1-C_5_ was more active under RL (IC_50_: 0.14 μm). In general, these PS could induce apoptotic cell death through lysosomes damage. The in vitro photosensitizing activity of the PS was not compromised after their immobilization onto graphene-based nanomaterials, with Zn-TMPyP@GQDs being the most promising hybrid system under RL (IC_50_: 0.37 μg/mL). Overall, our data confirm that GO and GQDs may represent valid platforms for PS delivery, without altering their performance for PDT on BC cells.

## 1. Introduction

Bladder cancer (BC) is the tenth most diagnosed type of cancer worldwide, with a higher occurrence in men [1]. It commonly begins as a non-muscle invasive form in 70% of cases, and it can be managed by complete transurethral resection, following repeated instillation of bacillus Calmette-Guerìn (BCG) [2]. However, cases of recurrence are not uncommon, and treatment options are limited. Moreover, recurrence of BC can evolve towards a more invasive form that can rapidly affect the muscular tissues (muscle-invasive BC, MIBC) and spread into metastasis [3]. Successful management of BC requires the combination of multiple therapeutic approaches, including chemotherapy and radiotherapy. These treatments often involve several serious side effects that can affect the patient’s life quality [4].

Among the new strategies designed for BC treatment, photodynamic therapy (PDT) seems to offer promising improved survival rates [5].

PDT relies on the simultaneous combination of three components: dioxygen (^3^O_2_), light, and a photosensitizer (PS) [6,7,8]. Upon light absorption, the PS is excited from its ground singlet state (S_0_) into a short-lived first excited singlet-state (S_1_), which undergoes intersystem crossing (ISC) towards an electronically excited triplet state (T_1_). When it returns to S_0_, it releases energy to ground state triplet dioxygen (^3^O_2_) to produce singlet oxygen (^1^O_2_) that, as other reactive oxygen species (ROS), is cytotoxic and promotes cellular death and tumor destruction [9,10]. An ideal PS for PDT should have the following characteristics: absorption in the 600–900 nm therapeutic window [11], minimal dark toxicity, solubility in body tissue fluids, photochemical reactivity with high triplet state yields, and long triplet state lifetime [12]. One of the major concerns of PDT is the appearance of several side effects, particularly with ALA-PDT, for the unspecific conversion of 5-ALA into protoporphyrin IX inside normal urothelium cells [13].

Porphyrins and their derivatives represent one of the major classes of PS for PDT [14,15]. Several attempts have been made to improve the efficiency of porphyrin-based PDT by conjugating porphyrin compounds to functionalized nanosystems (e.g., nanoparticles, liposomes, micelles, etc.) that may confer better PS solubility and selectivity to target tumor tissues [16,17]. Synergistic effects between porphyrin-like compounds and graphene-based nanomaterials in PDT have been reported, namely by using graphene oxide (GO) and graphene quantum dots (GQDs) as drug carriers [18,19,20,21,22,23]. For instance, these materials, besides offering a large surface area with reactive functional groups (which turns them into promising nanocarriers to transport and deliver photoactivatable drug molecules to tumor sites), can also diminish the aggregation tendency of drugs in physiological solution [24].

Furthermore, the release of drug molecules is facilitated in the diseased tissue as tumors have been demonstrated to exhibit acid pH values, while the pH of normal tissue is 7.4 [25,26].

The research here proposed reports the one-step preparation of GO and GQDs nanoplatforms loaded with three different tetracationic porphyrins to obtain efficient delivery systems.

The in vitro antiproliferative activity of our loaded nanoplatforms was investigated on the T24 human BC cell line, a high-grade and invasive transitional cell carcinoma selected as a BC model, along with their ability to generate intracellular ROS and the intracellular localization. Our idea for the therapeutical application of these nanoplatforms was to improve the solubility of the PS in saline solution for patient administration, to allow an efficient release of PS in cancer tissues, and to provide a multipurpose platform that can be easily functionalized with active-targeting molecules.

## 2. Materials and Methods

### 2.1. Syntheses of Tetracationic Porphyrins

The commercial reagents and solvents used for the organic syntheses were used as acquired (reagent grade purity) or were purified according to literature procedures [27]. Column chromatography (silica gel, 35–70 mesh, Merck, Darmstadt, Germany) or 20 × 20 cm preparative thin-layer chromatography (TLC) plates (coated with silica gel, 60 mesh, Merck, Darmstadt, Germany) were used to purify the reactional mixtures. ^1^H NMR and ^13^C NMR spectra were recorded on Bruker Avance 300 (300.13 MHz) and 500 (125.76 MHz) spectrometers, respectively (Bruker, Wissembourg, France). CDCl_3_, CD_3_OD, and DMSO-d_6_ were used as solvents, and tetramethylsilane (TMS) as an internal reference; the chemical shifts are expressed in *δ* (ppm) and the coupling constants (*J*) in Hertz (Hz). ESI-MS(+) spectra were recorded using a Micromass Q-Tof-2 spectrometer (Thermo Fisher Scientific, Manchester, UK), with CHCl_3_ or with a mixture of MeOH/formic acid (1%) as a solvent. High-resolution mass spectra (HRMS) were recorded on a LTQ Orbitrap XL mass spectrometer (Thermo Fisher Scientific, Bremen, Germany) using CHCl_3_ as a solvent. The full characterization (NMR, MS) is given in Supplementary Material.

#### 2.1.1. Synthesis of TMPyP

TMPyP was synthesized following previously described literature [28,29,30]. The neutral precursor 5,10,15,20-tetra(4-pyridyl)porphyrin (TPyP) was synthesized through the reaction of 4-pyridinecarboxaldehyde (5.5 mL, 58 mmol) with pyrrole (4.0 mL, 58 mmol) in refluxing acetic acid (120 mL) and nitrobenzene (70 mL) for 1 h at 120 °C. The solvents were distilled under reduced pressure. The crude reaction was then purified by column chromatography using a mixture of dichloromethane (DCM) and methanol (MeOH). TPyP was obtained after crystallization in DCM:MeOH (11% yield). The methylation of the pyridyl units in TPyP (30 mg, 4.80 × 10^−2^ mmol) was performed in the presence of a large excess of iodomethane (725 μL, 11.64 mmol) in DMF (1 mL) for 24 h at 40 °C. After this period, diethyl ether was added to precipitate the porphyrin. The compound was then filtered and washed with diethyl ether and finally crystallized in a mixture of DCM:MeOH (70% yield).

#### 2.1.2. Synthesis of Zn-TMPyP

Zn-TMPyP was synthesized directly from the free-base TMPyP. The complexation was accomplished through the reaction of TMPyP (50.0 mg, 4.21 × 10^−2^ mmol) with an excess of 1.5 equiv of Zn(II) acetate dihydrate (13.9 mg, 6.32 × 10^−2^ mmol) in a solution of CHCl_3_:MeOH (3:1) at 50 °C for 1.5 h. After the terminus of the reaction, the porphyrin was crystallized firstly in a mixture of propanol and diethyl ether, while a second crystallization step was carried out in a mixture of MeOH and hexane (90% yield).

#### 2.1.3. Synthesis of P1-C_5_

The synthesis of P1-C5 was based on previous literature [31,32]. The tetrabromination of 5,10,15,20-tetraphenylporphyrin (TPP, 100 mg, 0.163 mmol) at the *β*-pyrrolic positions was accomplished in the presence of 6 equiv of *N*-bromosuccinimide (NBS, 174 mg, 0.978 mmol) in CHCl_3_ (40 mL) at 60 °C for 24 h [33]. After the adequate work-up procedures (extraction, purification, and crystallization), the 2,3,12,13-tetrabromo-5,10,15,20-tetraphenylporphyrin (β-Br_4_TPP) was isolated (54% yield). The reaction of β-Br_4_TPP (60.8 mg, 6.54 × 10^−2^ mmol) with 1.5 equiv of Ni(II) acetate (17.3 mg, 9.81 × 10^−2^ mmol), at 140 °C for 1 h in DMF (2.5 mL), afforded the required Ni(II) complex (Ni-β-Br_4_TPP, 67% yield). The Heck coupling between Ni-β-Br_4_TPP (25.5 mg, 2.58 × 10^−2^ mmol) and 45 equiv of 4-vinylpyridine (123 μL, 1.16 mmol) was promoted using 0.5 equiv of Pd(OAc)_2_ (2.90 mg, 1.29 × 10^−2^ mmol), 1.3 equiv of PPh_3_ (8.79 mg, 3.35 × 10^−2^ mmol), and 3.75 equiv of K_2_CO_3_ (13.37 mg, 9.68 × 10^−2^ mmol), at 120 °C for 35 min under nitrogen atmosphere, in a mixture of DMF (0.5 mL) and toluene that had been passed through alumina (1 mL). After the work-up, the porphyrin was purified by TLC using a mixture of DCM:MeOH (4%), and the desired benzoporphyrin Ni-P1 was isolated (48% yield). The demetalation of this metalloporphyrin (13.4 mg, 1.24 × 10^−2^ mmol) was achieved in acidic conditions, using a chloroform solution (2 mL) with 10% of H_2_SO_4_ at room temperature for 1 h. After neutralization with sodium hydrogen carbonate, the product was washed with distilled water and extracted with DCM (52% yield). The quaternization of the pyridyl units in P1 (13.2 mg, 1.24 × 10^−2^ mmol) was performed in DMF (1 mL) at 40–80 °C for 24–72 h using a large excess of iodopentane (389 μL, 2.98 mmol). The tetracationic porphyrin bearing pentyl chains (P1-C_5_) was isolated from the reaction medium after precipitation with diethyl ether (74% yield).

### 2.2. Characterization of Porphyrin@GO and Porphyrin@GQDs Hybrids

The abovementioned porphyrins (TMPyP, Zn-TMPyP, and P1-C_5_) were non-covalently functionalized with GO (GO powder, Graphenea, San Sebastian, Spain) or GQDs (1.0 mg mL^−1^ dispersion of cyan luminescent GQDs in H_2_O, Merck, Darmstadt, Germany). The average thickness of the individual GO flakes was found to be 1.5–1.9 nm (3 layers) by atomic force microscopy (AFM), while for GQDs, the manufacturer indicated a 1–2.0 nm thickness (3–4 layers). UV-VIS absorption spectra were recorded on a Jasco V-560 spectrophotometer and a Varian Cary 50 spectrophotometer, using quartz cells (1 cm × 1 cm). Fluorescence spectra were recorded on Jasco FP-8300 and Varian Eclipse spectrofluorimeters, with excitation and emission slits set at 10 and 5 nm, respectively. Raman studies were performed in a combined Raman-AFM confocal microscope WITec alpha300 RAS+, through the excitation with a 532 nm line of a Nd:YAG laser (2 s, 10 acquisitions, 1 mW or 3 mW laser power for porphyrin@GO or porphyrin@GQDs, respectively). A Hitachi HD-2700 STEM microscope operating at 200 kV was employed to collect the STEM images on carbon-coated Cu grids.

The interactions between porphyrins and GO were monitored by spectrophotometric and spectrofluorimetric titrations at room temperature. Briefly, aliquots of a GO aqueous suspension (1.0 mg mL^−1^ or 3.0 mg mL^−1^) were successively added to an aqueous solution of porphyrin (2.0 μm or 6.0 μm). GO was added until three consecutive and similar measurement values of absorption red-shift and fluorescence were obtained, meaning that optimal interactions were achieved. In total, around 50 μL of GO was added to 1.0 mL of porphyrin. The synthesis of the porphyrin@GQDs hybrids was monitored oppositely, using the porphyrin solutions as a titrating agent (rather than the carbon nanomaterial), considering that GQDs have a stronger luminescence emission compared to porphyrins fluorescence. Several volumes of a porphyrin solution (stock 42 μm) were added to a solution of GQDs (0.1 μg mL^−1^). The fluorescence spectra of the hybrids were recorded at excitation at 330 nm, with 10 min delay between each addition. These spectrofluorometric titrations were further extended to other concentrations of porphyrin and carbon nanomaterials to allow the determination of the ideal binding ratio, which was used to prepare the treatment solutions for cellular assays. Porphyrins and graphene derivatives were mixed, considering the previously determined binding ratio, and placed on an orbital shaker for 30 min to allow the two components to interact. The solution was then diluted to a final 1.0 mg mL^−1^ concentration using PBS supplemented with 2% antibiotic/antimycotic solution and used to prepare the treatment solutions.

### 2.3. Light Exposure

Two irradiation devices were used for the irradiation of cell cultures during the assays: (i) a UVH436F medical lamp (Waldmann, Villingen-Schwenningen, Germany) was used for BL irradiation, with an irradiance of 17 mW cm^−2^ and a centered emission peak at 417 nm; (ii) a custom-build Oslon+ LED array system (Osram, Munich, Germany) was used to perform RL irradiation at an irradiance of 12 mW cm^−2^. The selected LED array emitted light with a narrow band, centered at 625 ± 20 nm. BL and RL irradiances were measured using a Waldmann Variocontrol radiometer, equipped with blue_v and PDT1200 sensors.

### 2.4. Cell Cultures and Cell Treatment

A T24 human bladder cancer cell line (ATCC/LGC Standards, Milan, Italy) was maintained in minimum essential media (MEM) culture medium (Life Technologies, Monza, Italy), supplemented with 10% FBS, 1% alanyl-glutamine, 1% non-essential amino acids, and 1% antibiotic/antimycotic solution (Sigma–Aldrich, Milan, Italy) at 37 °C, in a humidified atmosphere containing 5% CO_2_. Cells (5 × 10^4^) were seeded in 96-well plates in proliferation medium. After 24 h, cells were treated with increasing concentrations of non-immobilized porphyrins, porphyrin@GO, and porphyrins@GQD hybrids, in phenol red-free MEM containing 3% fetal bovine serum (FBS). After 1 h (non-immobilized porphyrins) and 4 h (porphyrin hybrids) of incubation, respectively, the medium was replaced with PBS and cells were treated with 2.5 J cm^−2^ of BL and 25.0 J cm^−2^ of RL. After irradiation, PBS was replaced with complete proliferation media. After 24 h, cell viability and cell death mechanisms were assessed. Dark control experiments were conducted, incubating cell cultures for 72 h with non-immobilized porphyrins or their hybrids with GO or GQDs, in complete proliferation media.

### 2.5. ROS and Singlet Oxygen Generation

The ROS assay was performed using the DCFDA—Cellular Reactive Oxygen Species Detection Assay Kit (Abcam, Cambridge, UK) according to the manufacturer’s instructions. Briefly, the cells (2.5 × 10^4^ cells/well) were seeded into each well of a 96-well clear bottom black plate (Corning, New York, NY, USA) in the proliferation medium. After 24 h, cultures were incubated with 20 μm 2′,7′-dichlorofluorescein diacetate (DCFDA) for 45 min and then treated with various concentrations of porphyrins/porphyrin hybrids for 1 h. Afterwards, the plate was irradiated with 2.5 J cm^−2^ of BL or 25.0 J cm^−2^ of RL. Data were collected using a Victor 3 multimodal microplate reader (λ_exc_/λ_em_ = 485/535). Cultures treated with 100 and 200 μm *tert*-butyl hydrogen peroxide (TBHP) were taken as positive controls.

Singlet Oxygen was determined using Singlet Oxygen Sensor Green, using a protocol developed by Gollmer et al. [34]. Briefly, cells (2.5 × 10^4^ cells/well) were seeded into each well of a 96-well clear bottom black plate (Corning, New York, NY, USA) in the proliferation medium. After 24 h, cells were washed with SMM (Standard Maintenance Medium, an aqueous solution of 140 mM NaCl, 3.5 mM KCl, 2 mM CaCl_2_, 2 mM MgCl_2_, 1.25 mM NaH_2_PO_4_, 10 mM glucose, and 10 mM HEPES, pH 7.35) and then incubated with 1 μm Singlet Oxygen Sensor Green (SOSG) for 2 h before being treated with various concentrations of porphyrins/porphyrin hybrids for 1 h. Afterwards, the plate was irradiated with 2.5 J cm^−2^ of BL or 25.0 J cm^−2^ of RL. Data were collected using a Victor 3 multimodal microplate reader (λ_exc_/λ_em_ = 485/535).

### 2.6. Cell Viability and Mechanisms of Cellular Death

Cell viability was assessed by resazurin assay. Briefly, cells were incubated with resazurin for 4 h, and the fluorescence of resulting resorufin produced by healthy cells was measured using a Victor 3 multimodal microplate reader (Perkin Elmer, Waltham, MA, USA). Data are expressed as mean ± the standard deviation (SD) of the mean. The difference between groups was evaluated using analysis of variance (ANOVA) with Graphpad Prism 9.0. Mechanisms of cell death were investigated by flow cytometry (BD FACSCanto II, BD, East Rutherford, NJ, USA) and the BD Pharmingen FITC Annexin V Apoptosis Detection Kit I, following the manufacturer’s indication. Briefly, cells were detached after 24 h after treatment, washed, and double-stained with Annexin V-FITC (AnnV-FITC, apoptosis marker) and 7-aminoactinomicyn D (7-AAD, necrotic marker) in the supplied binding buffer. Unmarked samples and samples individually marked with AnnV-FITC and 7-AAD were used for setting analysis parameters.

### 2.7. Intracellular Localization

T24 cells were grown on imaging dishes and, after 24 h, incubated at 37 °C with a final concentration of 10.0 μm of the three porphyrins and respective hybrids for 1 h and 4 h, respectively. Lysosomes were labeled using Lysotracker Green DND-26 (Life Technologies, Monza, Italy) for 15 min. Samples were imaged using an SP5 confocal laser scanning microscope (Leica, Wetzlar, Germany).

## 3. Results and Discussion

### 3.1. Synthesis and Characterization of the Tetracationic Porphyrins 

**Scheme 1:**
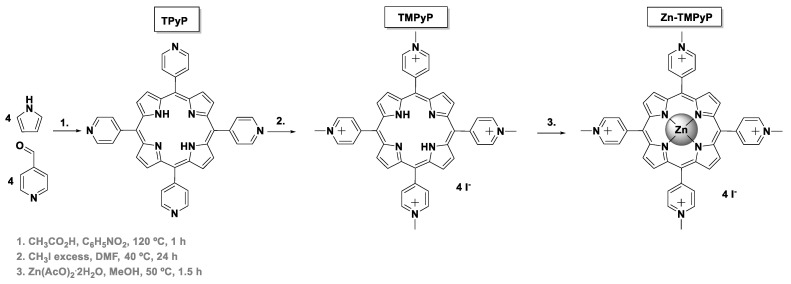
Synthesis of TMPyP and Zn-TMPyP.

**Scheme 2:**
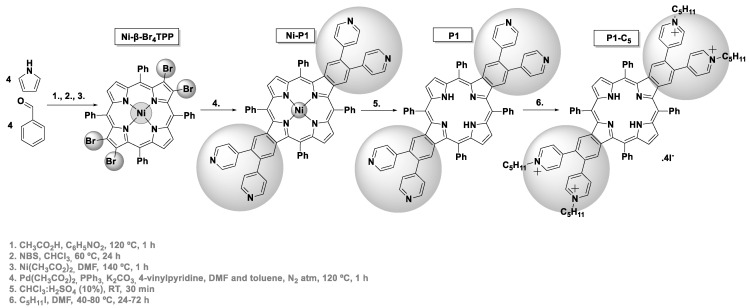
Synthesis of P1-C_5_.

The synthetic routes used to obtain the tetracationic porphyrins (TMPyP, Zn-TMPyP, and P1-C_5_) are summarized in Scheme 1 and Scheme 2. These three cationic porphyrins were selected because they are readily internalized by cells in comparison to the non-cationic counterparts, and their positively charged structure allows non-covalent interactions to both GO and GQDs at physiological pH.

Access to the cationic porphyrin TMPyP and to the Zn(II) complex (Zn-TMPyP) required the previous preparation of the neutral 5,10,15,20-tetra(4-pyridyl)porphyrin (TPyP), which was obtained in 11% yield via acid-catalyzed condensation of pyrrole with 4-pyridinecarboxaldehyde, according to well-established literature procedures (Scheme 1) [29,35]. The quaternization of TPyP pyridyl units with iodomethane then afforded the desired free-base TMPyP in 70% yield. The Zn-TMPyP was obtained in 90% yield after the core metalation of the free-base with Zn(II) acetate.

The synthesis of the tetracationic dibenzoporphyrin P1-C5 (Scheme 2) was based on the procedure described by Wang et al. for preparing this type of π-extended porphyrins [31,32], but using the 5,10,15,20-tetraphenylporphyrin (TPP) as a scaffold. The tetrabromination of this porphyrin at opposite β-pyrrolic positions and the core metalation with Ni(II) was followed by a four-fold Heck reaction between Ni-β-Br_4_TPP and 4-vinylpyridine. This synthetic step was carried out in a mixture of *N,N*-dimethylformamide (DMF) and toluene, using Pd(OAc)_2_ as a catalyst, PPh_3_ as a ligand, and K_2_CO_3_ as a base to afford the key intermediate Ni-P1 in 48% yield. The demetalation of this porphyrin was carried out in acidic conditions (in 52% yield), and then the quaternization of the pyridyl units from P1 was achieved with iodopentane to afford the final cationic porphyrin P1-C_5_ in 74% yield.

**Figure 1:**
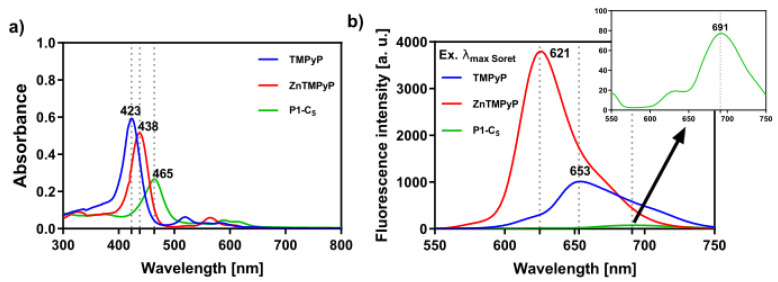
(**a**) UV-VIS absorption spectra of the studied porphyrins in PBS and (**b**) respective fluorescence spectra upon excitation at the wavelength of their Soret band ((TMPyP) = (Zn-TMPyP) = (P1-C_5_) = 6.0 × 10^−6^ M); TMPyP (λ_exc_ = 423 nm), Zn-TMPyP (λ_exc_ = 438 nm), and P1-C_5_ (λ_exc_ = 465 nm).

All porphyrin derivatives were characterized by nuclear magnetic resonance (NMR) spectroscopy, electrospray ionization mass spectrometry (ESI-MS), and UV-VIS spectroscopy (see Materials and Methods section and Figures S1–S9 in Supplementary Materials). In particular, the UV-VIS spectra of the porphyrins in phosphate buffer saline solution (PBS) show an intense Soret band (S_0_→S_2_ transitions), which peaked at 423 nm and 438 nm for TMPyP and Zn-TMPyP, respectively (Figure 1a); moreover, the Q bands (S_0_→S_1_ transitions) lie between 550 and 650 nm. The Soret band of the P1-C_5_ porphyrin is red-shifted (to 465 nm) in comparison to TMPyP and Zn-TMPyP due to extensive electronic delocalization of the aromatic structure, which lowers the required energy for electronic transitions from the fundamental state to the excited states of the porphyrin. The molar absorption coefficients have been calculated at the maximum absorption of the three porphyrins (Table 1), as well as at the wavelength emission of the two lamps used for the visible light irradiation in the biological experiments (417 nm for BL and 625 nm for RL). Furthermore, the three porphyrins show fluorescence emission maxima between 600 and 750 nm (Figure 1b).

### 3.2. Characterization of Neat Carbon Nanomaterials

The commercial GO and GQDs selected to prepare the new hybrids with the cationic porphyrins were characterized by different techniques (UV-VIS, fluorescence and Raman spectroscopies, and electron microscopy) in order to evaluate the influence of the structural composition and morphological features in the electronic and optical properties of the final hybrid nanomaterials.

**Figure 2:**
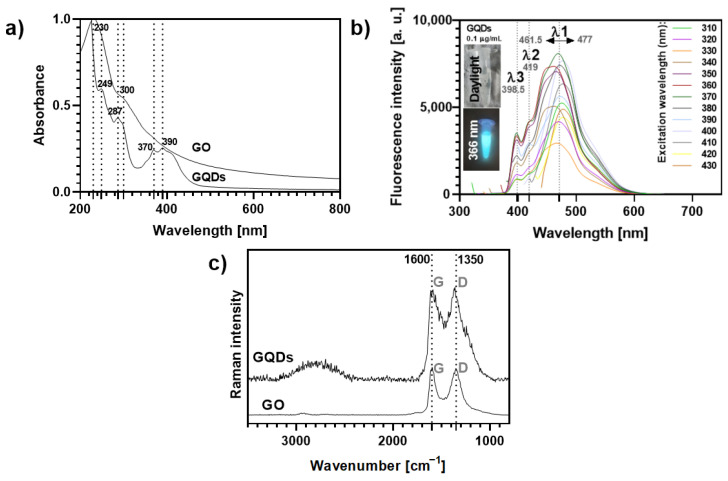
(**a**) UV-VIS absorption spectra (normalized at 230 nm) of aqueous solutions of GO and GQDs. (**b**) Variation of the fluorescence profile of GQDs 0.1 μg mL^−1^ according to the excitation wavelength (ranging from 310–430 nm). The inset photos show the blue fluorescence of the GQDs solution (0.1 μg mL^−1^) observed under a 366 nm lamp as compared to its optical transparency under daylight conditions. (**c**) Raman spectra of GO and GQDs (532 nm excitation).

Both carbon nanomaterials absorb strongly in the UV region (Figure 2a). GO displays two main peaks around 230 nm (π-π* electronic transitions of the graphitic *sp*^2^ domains), with a shoulder around 300 nm (n-π* transitions), while for GQDs such electronic transitions originate absorption bands peaked at 249 nm and had a shoulder at 287 nm. In addition, a typical broad absorption band with peaks around 370 and 390 nm can be detected in the absorption spectrum of GQDs, which is ascribed to the absorption of different surface functional groups [36,37,38].

The GQDs solutions exhibit a bright cyan-blue fluorescence under UV light irradiation (insets Figure 2b) at a concentration as low as 0.1 µg mL^−1^, while GO aqueous suspensions do not show fluorescence in these conditions (Figure S10). As highlighted in Figure 2b, the fluorescence intensity and maximum emission wavelength of GQDs at this concentration depend on the excitation wavelength (λ_exc_, 310–430 nm). It has been reported that excitation-dependent emission fluorescence in GQDs may be ascribed to particle size effects and their surface properties. This is in line with changes in the location of the main emission peak, λ1, which shows a 15.5 nm shift, depending on the λ_exc_. The shifts observed for the other emission peaks (λ2~419 nm and λ3~398.5 nm) are negligible. The maximum intensity for λ1 was recorded at λ_exc_ = 370 nm, while the minimum intensity occurred at λ_exc_ = 330 nm.

Two strong Raman bands were observed for GO and GQDs (Figure 2c): (i) the G band (*ca*. 1600 cm^−1^), characteristic of a carbon network with *sp*^2^ hybridization, arising mainly from the in-plane C-C deformations; (ii) the disorder-induced D band (around 1350 cm^−1^), activated by symmetry breaking at defects and edges of the graphene lattice, namely oxygen groups on *sp*^3^-carbon moieties [39,40,41]. Moreover, both GO and GQDs show a broad feature with low intensity at about 2500–3100 cm^−1^, corresponding to 2D and combination bands, which is more pronounced for the GQDs sample, indicating a multilayered and disordered arrangement of the carbon nanosheets [38,41,42]. The average thickness of the individual GO flakes was found to be 1.5–1.9 nm (3 layers) by atomic force microscopy (AFM, Figure S11), while for GQDs the manufacturer indicated a 1–2.0 nm thickness (3–4 layers) [42,43,44].

The morphology of the carbon nanomaterials was analyzed by scanning transmission electron microscopy (STEM, Figure S11). GO sheets are observed as thin folded sheets of low contrast, while GQDs appear as quasi-spherical nanoparticles with higher contrast. The average diameter of the GQDs (26.0 ± 3.8 nm), measured as the circles circumscribing the dots, was larger than expected (~10 nm). This might be due to the coalescence of the GQDs that had probably occurred to some extent during the STEM analysis.

### 3.3. Synthesis and Characterization of Non-Covalent Hybrids of Porphyrin@Carbon Nanomaterials

Herein, we report the multifunctional hybrids obtained from each tetracationic porphyrin and the nanomaterials GO and GQDs. The interactions between both components were monitored by spectrophotometric and spectrofluorometric titrations at room temperature, and the ensuing hybrids porphyrin@GO and porphyrin@GQDs (where porphyrin stands for TMPyP, Zn-TMPyP, or P1-C_5_) were characterized by Raman spectroscopy and electron microscopy.

#### 3.3.1. Porphyrin@GO Hybrids

**Figure 3:**
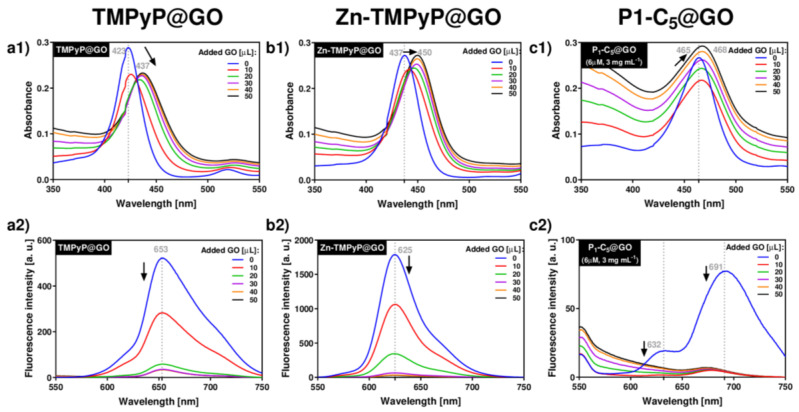
UV-VIS (**a1**–**c1**) and fluorescence (**a2**–**c2**) spectra corresponding to the titrations of each porphyrin with GO suspensions in PBS medium: TMPyP (**a1**,**a2**) and Zn-TMPyP (2.0 μm, **b1**,**b2**) with GO 1.0 mg mL^−1^; P1-C_5_ (6.0 μm) with GO 3.0 mg mL^−1^ (**c1**,**c2**).

The spectrophotometric and spectrofluorometric titrations of porphyrins with GO typically led to a red-shift of the porphyrin’s absorbance and the quenching of its original fluorescence (Figure 3).

Upon addition of successive aliquots of GO to TMPyP, the original Soret band of TMPyP (λ_max_ 423 nm) was red-shifted to 437 nm (Δλ 14 nm), and its absorption intensity decreased (Figure 3(a1)). In addition to these changes, a decrease of the porphyrin emission band intensity at 653 nm was observed (Figure 3(a2)). These observations support the formation of TMPyP@GO hybrids through non-covalent interactions caused by the molecular flattening of the four cationic methylpyridinium moieties of the porphyrin molecules onto the GO sheets [45,46].

Zn-TMPyP displayed a slightly lower red-shift of the Soret band (from 437 nm to 450 nm, Δλ 13 nm) when compared to TMPyP, and the final absorbance of the hybrid material did not decrease (Figure 3(b1)). Among the porphyrins, Zn-TMPyP is more fluorescent than TMPyP, but the addition of small aliquots of GO suspension caused a pronounced quenching effect in both systems (Figure 3(b2)). The strong affinity of Zn-TMPyP towards GO may have the contribution of the coordination of Zn(II) to oxygen groups present at the GO surfaces [47].

In order to perform the experiments in similar absorbance ranges, the concentrations of P1-C_5_ and GO were increased, respectively, to 6.0 μm and 3.0 mg mL^−1^, thus maintaining the ratio of the respective amounts used in the previous studies. The addition of GO to P1-C_5_ caused a small red-shift (from 465 to 468, Δλ 3 nm) of the Soret band (Figure 3(c1)), but the fluorescence was also quenched (Figure 3(c2)). The major spectroscopic differences observed in the titration of P1-C_5_ in relation to the other charged porphyrins investigated can be explained by its chemical structure, which contains two fused aromatic rings bearing the positively charged pyridyl units at opposite *β*-positions and phenyl rings at the *meso*-positions. One possible explanation for the negligible UV-VIS changes after GO addition is that P1-C_5_ is more planar than TMPyP and its substituents are not rotated to lie flat in the GO as they do in TMPyP.

**Figure 4:**
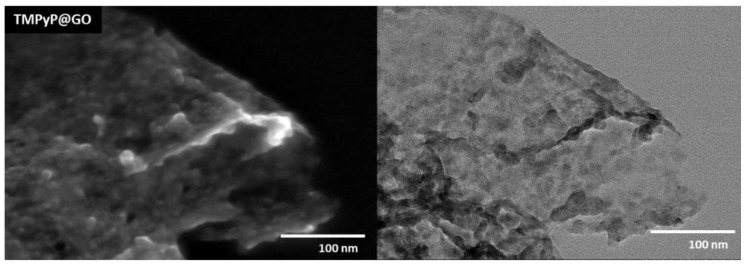
STEM images of the hybrid TMPyP@GO, which was obtained from non-covalent functionalization of GO using the porphyrin TMPyP.

After the non-covalent functionalization with porphyrins, the GO sheets became wrinkled and decorated with darker flakes, which presumably correspond to over-stacked porphyrin layers, as illustrated in Figure 4 for the TMPyP@GO sample. The STEM images suggest that the whole surface of GO sheets is covered by such porphyrin layers, although there is a tendency for the accumulation of porphyrins at the edges or wrinkles of GO sheets. This can be explained because the aromatic groups in the GO sheets induce a homogeneous distribution of the porphyrins over the GO surfaces through π-π stacking, but still, there is a preferential distribution of porphyrin through electrostatic interactions between the deprotonated carboxyl groups at the edges of GO structure and the positively charged pyridyl groups of the porphyrin.

**Figure 5:**
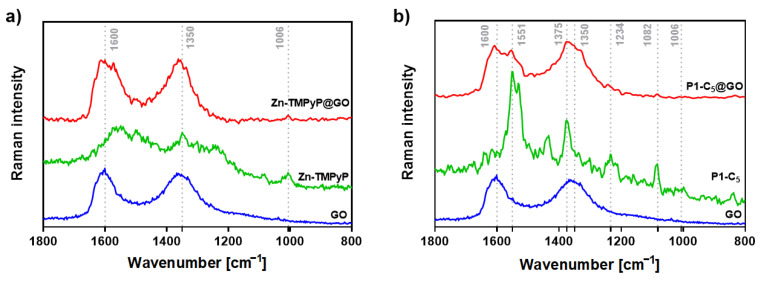
(**a**) Raman spectra (532 nm excitation) of free GO (blue line), Zn-TMPyP (green line), and Zn-TMPyP@GO (red line) and (**b**) corresponding data for the studies with P1-C_5_ and GO.

Several Raman studies were carried out in order to assess the above hypothesis on the type of chemical interactions underlying the formation of the hybrids (Figure 5). We have previously demonstrated that the binding of TMPyP to the edges of GO causes an intensification of the porphyrin’s Raman band at 978 cm^−1^ in the TMPyP@GO hybrids, associated with an in-plane bending of the positively-charged pyridyl groups (Figure S12) [45]. Thus, TMPyP is attached to the edges of GO, mostly via electrostatic interactions involving the methylated pyridines and the deprotonated carboxyl groups of GO.

On the other hand, the Zn-TMPyP@GO hybrid (Figure 5a) shows a vibrational mode around 1006 cm^−1^, which corresponds to the vibration of the porphyrin’s core. This suggests that the interaction between Zn-TMPyP and GO occurs mainly through the porphyrin core.

To the best of our knowledge, there are no Raman assignments of the vibrational modes for P1-C_5_ porphyrin. Still, in the P1-C_5_@GO hybrid (Figure 5b), both the D and G bands of GO are broadened and more well-resolved, containing additional peaks that might be ascribed to the vibrational modes of individual P1-C_5_, proving the successful interaction between the two components of the hybrid.

#### 3.3.2. Porphyrin@GQDs Hybrids

**Figure 6:**
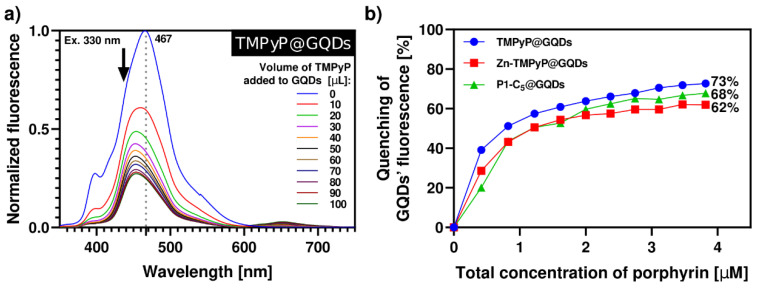
(**a**) Normalized fluorescence spectra of aqueous solution of TMPyP@GQDs upon excitation at 330 nm with 10 min delay between each measurement. The spectra were obtained by successive additions of the porphyrin solution (42 µM) to a solution of GQDs 0.1 µg mL^−1^. (**b**) Quenching of GQDs’ fluorescence caused by the addition of porphyrins in each system of porphyrin@GQDs (λ_exc_ = 330 nm). The inset highlights the loss of GQDs’ fluorescence in the final hybrid solutions under a 366 nm lamp.

The synthesis of porphyrin@GQDs hybrids was monitored by optical measurements, as noted above, but unlike for the GO system, aliquots of the porphyrin solution were added to the GQDs solution (see Experimental section). This alteration was required because the GQDs show more intense luminescence as compared to the porphyrins. These studies were monitored by spectrofluorometric titrations, as shown in Figure 6; note that the GQDs (0.1 µg mL^−1^) suspensions show low absorbance, which precluded the monitoring by spectrophotometric titrations (Figure S13). All the samples have been excited at 330 nm with 10 min breaks between each measure to promote the solutions’ stability and decrease the effect of GQDs’ self-quenching through time (26%, Figure S14). In all cases, the porphyrins outweighed the self-quenching of GQDs (Figure 6). By increasing the total porphyrin concentration up to 4.0 µM, TMPyP causes a 73% quenching of the initial GQDs’ fluorescence, followed by P1-C_5_ (68%) and Zn-TMPyP (62%). This occurs because the absorption bands of the porphyrins (Figure 1) partially overlap with the emission bands of the GQDs at the selected excitation wavelength; therefore, the inner filter effect of porphyrins on GQDs results in substantial fluorescence quenching of the GQDs throughout the titrations, particularly for TMPyP.

**Figure 7:**
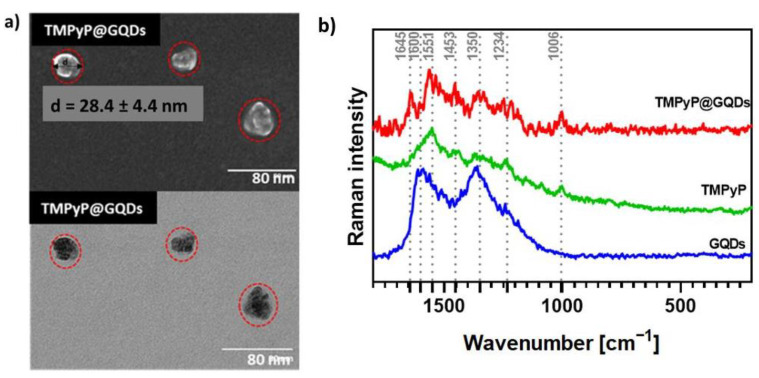
(**a**) STEM images of TMPyP@GQDs. The red dotted lines represent the circles circumscribing the TMPyP@GQDs hybrids observed in the microscopy analysis. (**b**) Raman spectra (532 nm excitation) of free GQDs (blue line), TMPyP (green line), and TMPyP@GQDs (red line).

The conjugation of GQDs with porphyrins led to a slight increase of the particle average size but still within a comparable size range, as exemplified in Figure 7a (28.4 ± 4.4 nm) for the TMPyP@GQDs hybrids. The Raman spectrum of the TMPyP@GQDs hybrid shows features that are also observed in the corresponding spectra of the free samples of GQDs and TMPyP (Figure 7b). However, the G band peak assigned to the GQDs is shifted to 1645 cm^−1^ in relation to the free sample (1600 cm^−1^), while the D band (1350 cm^−1^) is peaked at the same wavenumber as the original GQDs sample. Additionally, some Raman bands ascribed to the porphyrin seem enhanced in the hybrid, as compared to the free sample, such as the bands at: 1006 cm^−1^ (stretching mode involving the *α*- and *meso*-carbons of the porphyrin core, ν C_α_-C_m_); 1551 cm^−1^ (ν C_β_-C_β_); and 1453 cm^−1^ (ν C_α_-C_β_). These observations suggest that the TMPyP molecules are associated with the GQDs by non-covalent interactions. A similar interpretation can be extended to the Raman data obtained for the other two hybrids (Zn-TMPyP@GQDs and P1-C_5_@GQDs, Figure S15).

### 3.4. Biological Experiments

#### 3.4.1. Photo-Antiproliferative Activity of non-Immobilized Porphyrins on T24 Human Bladder Cancer Cells

Before testing the photo-antiproliferative activity of the non-immobilized porphyrins, their photostability was investigated under increasing irradiations of both RL (0–35 J cm^−2^) and BL (0–20 J cm^−2^) lights. While TMPyP and Zn-TMPyP proved to be photostable under both light irradiations (data not shown), P1-C_5_ demonstrated a high instability under both light irradiations, as shown by the progressive decrease of its absorption spectrum during irradiation (Figure S16). Still, none of the spectra evidenced an isosbestic point, which suggested the absence of stable products formed during the photodegradation process.

The light doses selected for irradiating the T24 cells were 2.5 J cm^−2^ for BL and 10 times higher for RL (25.0 J cm^−2^); indeed, the higher RL dose was necessary for the activation of the compounds, in line with their molar absorption coefficients at 417 nm and 625 nm (Table 1). Moreover, both light doses were chosen as non-toxic for the cells when applied alone (without any porphyrin or porphyrin@GO/GQDs, data not shown).

**Figure 8:**
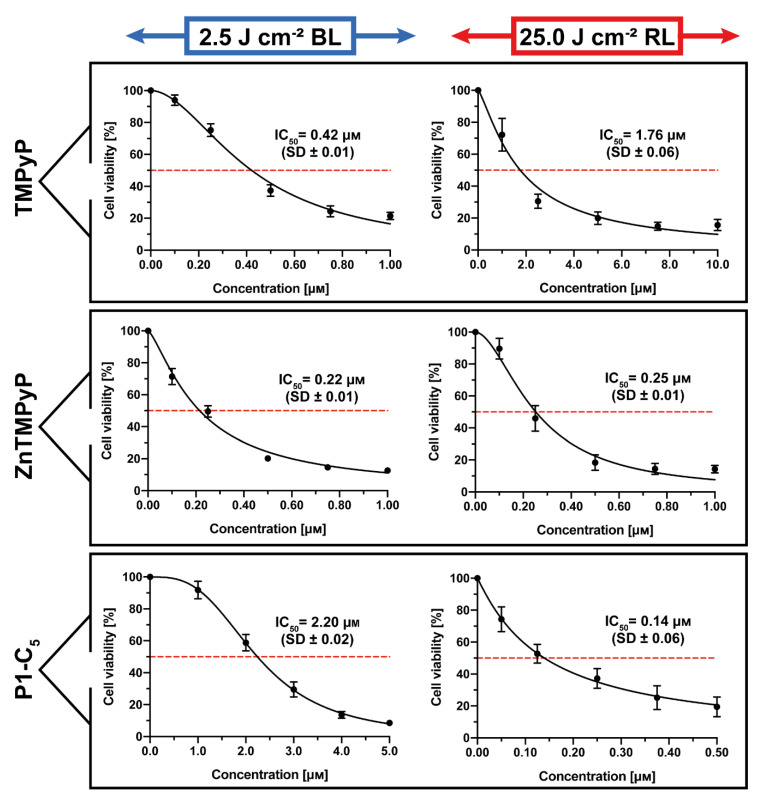
Antiproliferative activity of TMPyP (**top**), Zn-TMPyP (**center**), and P1-C_5_ (**bottom**), incubated for 1 h after irradiation with 2.5 J cm^−2^ of BL (**left**) and 25 J cm^−2^ of RL (**right**) on T24 human BC cells. Data are expressed as mean ± SD of at least three independent experiments carried out in triplicate.

The obtained results under both irradiations when the T24 human BC cells were incubated for 1 h with the three cationic porphyrins are represented in Figure 8.

TMPyP showed significant antiproliferative activity against T24 human BC cells (Figure 8, top) following irradiation with BL (IC_50_ 0.42 ± 0.01 μm) and under RL (IC_50_ 1.76 ± 0.06 μm).

The Zn(II) analogue Zn-TMPyP instead demonstrated strong and similar photo-antiproliferative activity under both light irradiations (Figure 8, center): IC_50_ of 0.22 ± 0.01 μm with BL and 0.25 ± 0.01 μm with RL. Note that, under both treatments, the concentration range used was lower (0–1.0 μm).

Finally, P1-C_5_ proved to be more active under red light (Figure 8, bottom): according to its absorption spectrum, this effect was higher under RL (IC_50_ of 0.14 ± 0.06 μm) at the concentration range of 0–0.5 μm, while the IC_50_ under BL was 2.20 ± 0.02 μm at the concentration range of 0–5.0 μm.

As noted previously, P1-C_5_ has been shown to be photo-unstable at increasing red and blue light doses (Figure S16), although its photodegradation seems to proceed without the formation of stable photoproducts. Considering the doses of BL and RL used for the experiments on cells, its instability under 25.0 J cm^−2^ of RL could be noteworthy for interpreting the in vitro experiments on cells. Therefore, to evaluate the potential role of any formed photoproduct in P1-C_5_ cellular action, this porphyrin was dissolved in PBS, then preirradiated with RL at a total light dose of 25.0 J cm^−2^ (as this light dose induced a marked photodegradation), and subsequently incubated with cells at the same concentration range used for non-preirradiated porphyrin. The preirradiated P1-C_5_ solution did not show any cytotoxic activity (see cell viability profile in Figure S17), confirming that the high photodynamic activity of the porphyrin P1-C_5_ is not associated with any photodegradation products.

#### 3.4.2. Detection of Singlet Oxygen and Other ROS in Cell Cultures

**Figure 9:**
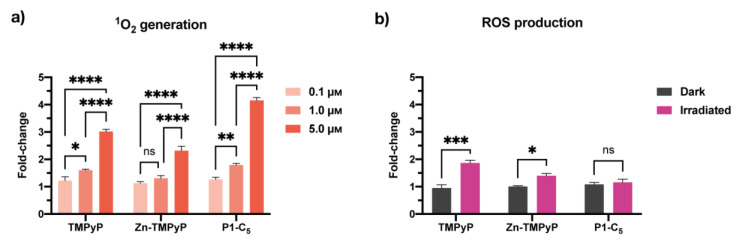
(**a**) Singlet oxygen production of non-immobilized porphyrins at concentrations 0.1 μm, 1.0 μm, and 5.0 μm under RL in T24 cells. (**b**) ROS production by the non-immobilized porphyrins tested at their IC_50_ under RL. Data are expressed as mean ± SD of at least two independent experiments carried out in triplicate. (* *p* < 0.0332, ** *p* < 0.0021, *** *p* < 0.0002, **** *p* < 0.0001; two-way ANOVA).

The production of singlet oxygen (^1^O_2_) by the porphyrins in T24 cell cultures was quantified under both BL and RL irradiations using Singlet Oxygen Sensor Green (SOSG) [34]. The ^1^O_2_ production was found to be proportional to the concentration of the compounds in the treated cells under RL (Figure 9) and BL (Figure S18). The amount of ^1^O_2_ correlates very well with their antiproliferative activity; in fact, P1-C_5_, which was able to produce more ^1^O_2_ under RL (Figure 9a), showed the lowest IC_50_ compared to the other porphyrins. Even under BL, there was correspondence between the porphyrin with the lowest IC_50_ (Zn-TMPyP) and the highest ^1^O_2_ production at all tested concentrations (Figure S19a).

2′,7′-dichlorofluorescein diacetate (DCFDA) was used to detect the formation of total ROS. When irradiated with RL, the production of ROS by TMPyP and Zn-TMPyP was about 1.5 times higher than the controls (cells irradiated with RL alone or cells incubated with porphyrins without irradiation), as shown in Figure 9b. On the other hand, P1-C_5_ did not seem to generate significant ROS concerning the reference samples.

Overall, the photo-antiproliferative activity of non-immobilized porphyrins irradiated with RL in T24 cells was not strictly correlated to the production of ROS. In fact, P1-C_5_, the most active porphyrin in killing T24 cells under RL, did not produce a significant amount of ROS, while TMPyP, the less active PS under RL, produced more ROS inside the cells than the other PS under study. Instead, BL (Figure S18b) induced the production of more ROS by the compounds; in particular, P1-C_5_ demonstrated higher amounts than the other two porphyrins and four times higher than the controls. This result suggests that such ROS are not the primary cause of photodamage, probably because the cells have defense mechanisms to destroy them, which does not occur for ^1^O_2_.

#### 3.4.3. Flow Cytometric Analysis

To investigate the type of cell death involved in the treatments with non-immobilized porphyrins, flow cytometric analysis of cell death mechanism was performed. Before flow cytometric analysis, T24 cells were previously treated with the three porphyrins at a concentration corresponding to their IC_50_, and then they were irradiated with RL at a light dose of 25 J cm^−2^.

**Figure 10:**
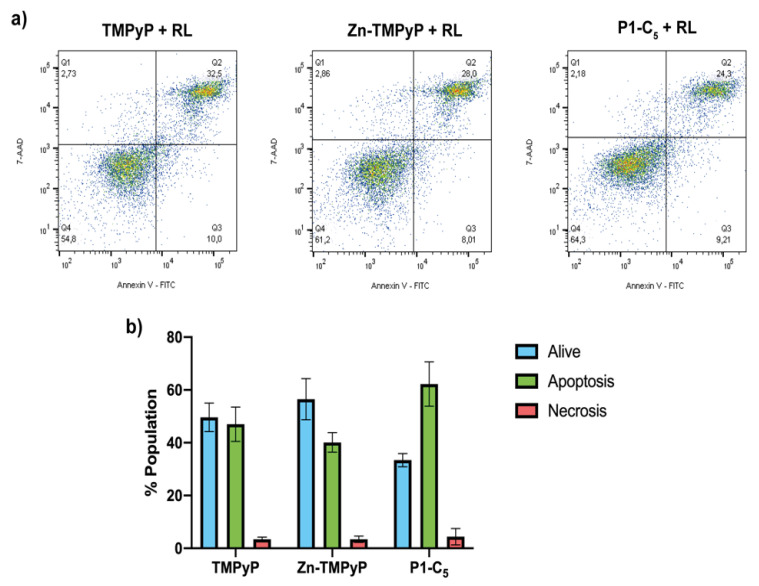
(**a**) Flow cytometric analysis of the cell samples treated for 1 h with TMPyP (left), Zn-TMPyP (center), and P1-C_5_ (right) irradiated with RL (25.0 J cm^−2^) and marked with Annexin V-FITC and 7-AAD. (**b**) Prevailing mechanism of cellular death after the treatment with each porphyrin. Data are expressed as mean ± SD of at least three independent experiments carried out in triplicate.

As can be seen from the right side of Figure 10a, for the sample treated with 1.76 μm TMPyP, the population found in the quadrant Q3 (corresponding to the living cells) was about 50% of the total population present, while the rest was mainly distributed in quadrants Q2 and Q4, thus indicating cell death due to apoptosis.

In the case of the sample treated with 0.25 μm of Zn-TMPyP, about half of the tested population appeared to be alive, while the remaining part was divided into quadrants Q2 and Q4 as TMPyP, thus indicating an apoptotic type of death (Figure 10a, center).

For P1-C_5_, although the concentration corresponding to its IC_50_ 0.14 μm was also used, the two-dimensional graph shows a slight reduction of the population in Q3, corresponding to the living cells; this result seems in contrast to the viability test with resazurin, but this could be explained by the greater sensitivity of the flow cytometric analysis concerning the viability test or to a late cellular damage due to a prolonged time between the marking and the reading at the flow cytometry. The remaining population is divided between quadrants Q2 and Q4, indicating death by apoptotic mechanism (Figure 10a, right).

This analysis clarifies the prevailing mechanism of apoptotic cell death induced by the three porphyrins under examination (Figure 10b).

#### 3.4.4. Photo-Antiproliferative Activity of Porphyrin@Carbon Nanomaterials on T24 Cells

##### Preparation of Porphyrin@GO and Porphyrin@GQD Hybrids for Cellular Experiments

**Figure 11:**
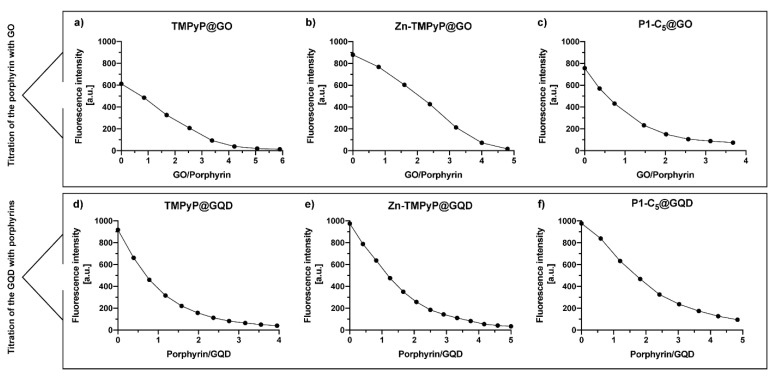
Spectrofluorometric titration of (**a**) TMPyP (2.0 μm), (**b**) Zn-TMPyP (2.0 μm), and (**c**) P1-C_5_ (6.0 μm) with a 2.0 mg mL^−1^ suspension of GO (λ_exc_ corresponds to the wavelength of the Soret band of each porphyrin). Fluorometric titration of GQDs (2.0 μg mL^−1^) with (**d**) TMPyP, (**e**) Zn-TMPyP, and (**f**) P1-C_5_. (λ_exc_ 497 nm).

To assess the performance of GO and GQDs as nanocarriers for the cationic porphyrins and to maximize their loading capability, an estimation of the saturation binding ratio between each porphyrin and the carbon nanomaterials was performed through spectrofluorimetric titrations (Figure 11; see details in the Experimental section). The binding ratios of the three porphyrin@GO hybrids were determined graphically (Figure 11a–c) by interpolating the obtained curves with two broken lines; the intersection between the two lines represents the binding ratio (Table 2). A similar approach was performed to determine the binding ratio of porphyrins@GQDs (Figure 11d–f) but, as mentioned above, these titrations were performed oppositely. The porphyrin solutions were used as a titrating agent rather than the GQDs due to their stronger luminescence emission when compared to porphyrins fluorescence; the titration of a porphyrin solution with GQDs resulted in the immediate detector signal saturation. The determined binding ratios are summarized in Table 2; the values of the binding ratio of porphyrin/GO varied between 1/4.2 and 1/2, while the binding ratio of porphyrin/GQDs varied between 2/1 and 3/1.

#### 3.4.5. Photo-Antiproliferative Activity of Porphyrin@GO Hybrids

**Figure 12:**
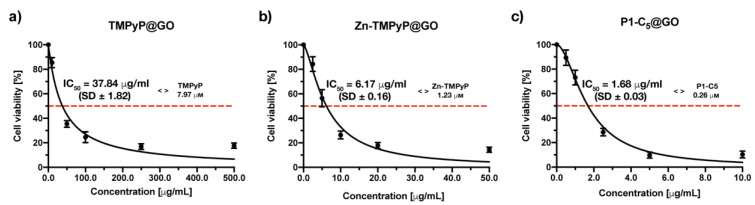
Antiproliferative photodynamic activity at different concentrations of (**a**) TMPyP@GO, (**b**) Zn-TMPyP@GO, and (**c**) P1-C_5_@GO after 4 h of incubation followed by irradiation with RL at a total light dose of 25 J cm^−2^. Data are expressed as mean ± SD of at least three independent experiments carried out in triplicate.

The in vitro tests on cells carried out to verify the photo-antiproliferative activity of the porphyrin@GO hybrids after irradiation with RL (Figure 12) followed the same procedure as with non-immobilized porphyrins, but the incubation time was increased up to 4 h to allow both the cell uptake of porphyrin@GO and/or porphyrin release from the hybrids.

The photodynamic activity of the TMPyP@GO hybrid was assessed at increasing concentrations (0–500.0 μg mL^−1^) (Figure 12a). The measured IC_50_ of the TMPyP@GO hybrid was 37.84 ± 1.82 μg mL^−1^, corresponding to a TMPyP concentration of 6.64 μm, a value significantly higher than the IC_50_ before its immobilization (1.76 μm).

The Zn-TMPyP hybrid was tested in a concentration range of 0–50.0 μg mL^−1^, ten times lower than that used for TMPyP@GO, which allowed identification of the IC_50_ of 6.17 ± 0.16 μg mL^−1^ (Figure 12b). The molarity of Zn-TMPyP corresponding to this concentration was 0.94 μm, approximately 4 times higher than the IC_50_ of non-immobilized porphyrin (0.25 μm).

When the cells were treated with increasing concentrations of P1-C_5_@GO hybrid, the obtained IC_50_ was 1.68 ± 0.03 μg mL^−1^, corresponding to a concentration of the non-immobilized porphyrin of 0.31 μm (Figure 12c). This value was only slightly higher than the IC_50_ of the non-immobilized P1-C_5_ (0.14 μm), thus the photodynamic activity of both non-immobilized and immobilized porphyrin is comparable.

#### 3.4.6. Photo-Antiproliferative Activity of Porphyrin@GQDs Hybrids

A previous check of GQDs cell toxicity-biocompatibility was performed before evaluating the photo-antiproliferative activity of the porphyrin@GQDs hybrids (Figure S19). The photocytotoxicity of non-functionalized GQDs against T24 cells was assessed in a wide range of concentrations (0–500.0 μg mL^−1^) as GQDs can produce singlet oxygen *per se*, without being charged with the PS, thus causing “independent” cytotoxic effects upon irradiation. The cells were incubated for 4 h with increasing concentrations of GQDs and were then irradiated with RL. GQDs were shown to be phototoxic at high concentrations, with an IC_50_ of 14.96 ± 0.94 μg mL^−1^; therefore, the concentration of GQDs present in the hybrids was lower than the IC_50_ identified by this test.

**Figure 13:**
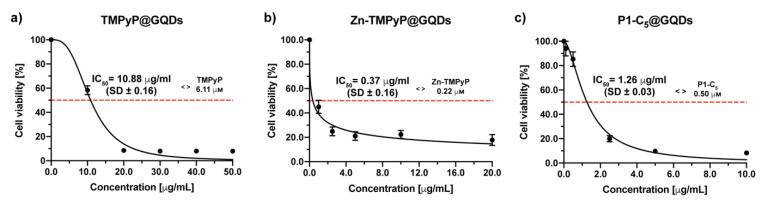
Antiproliferative photodynamic activity at different concentrations of (**a**) TMPyP@GQDs, (**b**) Zn-TMPyP@GQDs, and (**c**) P1-C_5_@GQDs on T24 cells after 4 h of incubation followed by irradiation with RL and a total light dose of 25 J cm^−2^. Data are expressed as mean ± SD of at least three independent experiments carried out in triplicate.

To evaluate the photo-antiproliferative activity of the porphyrin@GQDs on T24 cells, the same procedure used for the non-immobilized porphyrins was followed. By using a concentration range of 0–50 μg mL^−1^, TMPyP@GQDs showed an IC_50_ of 10.88 ± 0.16 μg mL^−1^ after irradiation with RL (Figure 13a). At this IC_50_, the TMPyP concentration in the hybrid corresponded to 6.11 μm, again a value significantly higher than the IC_50_ before its immobilization (1.76 μm).

The Zn-TMPyP@GQDs hybrid has been tested in the concentration range of 0–20 μg mL (Figure 13b), and the IC_50_ was 0.37 ± 0.06 μg mL^−1^, corresponding to a porphyrin concentration of 0.22 μm, was slightly lower than that identified for the non-immobilized Zn-TMPyP (0.25 μm).

P1-C_5_@GQDs has also demonstrated a high cytotoxic activity (concentration range 0–10 μg mL^−1^) with an IC_50_ of 1.26 ± 0.03 μg mL^−1^, corresponding to a porphyrin concentration of 0.50 μm (approximately 3 times higher than the IC_50_ of the non-immobilized P1-C_5_, 0.14 μm) (Figure 13c).

#### 3.4.7. Intracellular Localization

To determine the T24 intracellular distribution of the three porphyrins in the free form and in the GO and GQD hybrids, confocal microscopy was used to visualize the intrinsic fluorescence of the compounds (porphyrin channel) along with LysoTracker Green DND-26, a marker for acidic organelles such as early endosomes and lysosomes, both as individual channels and merged.

**Figure 14:**
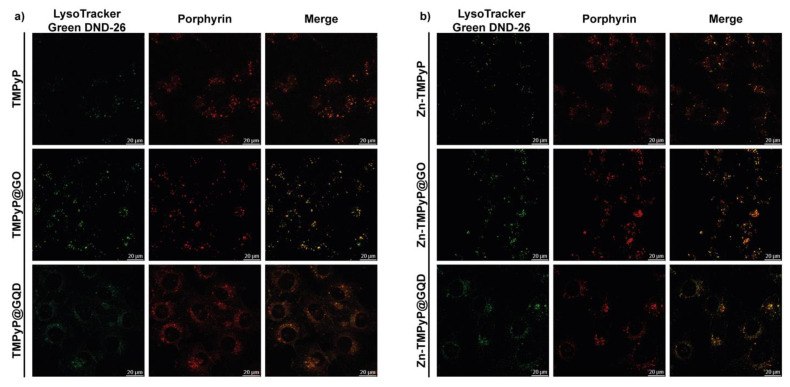
Intracellular localization of TMPyP (**a**) and Zn-TMPyP (**b**) in free form (top) and hybridized with GO (center) and GQD (bottom).

As shown in Figure 14, high co-localization (yellow) of the porphyrin red fluorescence with the green fluorescence of LysoTracker demonstrated that TMPyP and Zn-TMPyP in the free form are primarily transported to the lysosomes. The same was observed with the GO and GQD hybrids. It must be pointed out that large aggregates are present outside the cells for GO hybrids, indicating the difficulty of these hybrids in being fully internalized. However, the lysosomal localization of the two porphyrins indicates that they can be released from the GO platform in a high enough concentration to carry out the photosensitizing activity of the drugs.

**Figure 15:**
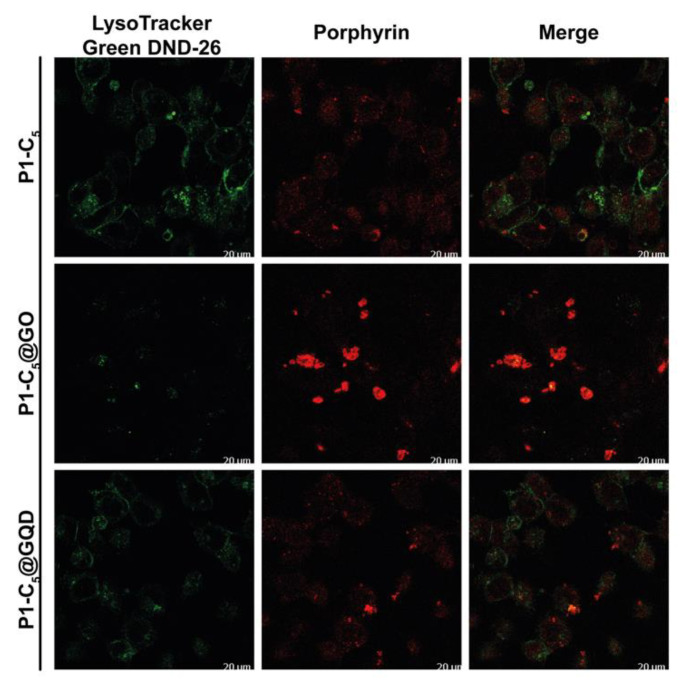
Detection of P1-C_5_ in the free form (**top**) and hybridized with GO (**center**) and GQDs (**bottom**).

Conversely, the distribution of free P1-C_5_ was not confined to specific organelles, and its signal did not co-localize with organelle-specific probes (Figure 15). Furthermore, such behavior has also been observed in the corresponding hybrids. The corresponding antiproliferative activity showed their efficacy, even without specific cell localization and damage.

## 4. Conclusions

The evaluation of the photo-antiproliferative activity of the tetracationic porphyrins (TMPyP, Zn-TMPyP, and P1-C_5_) before and after non-covalent immobilization onto GO and GQDs proved their potential as photosensitizers towards T24 human BC cells under different light irradiations. The photodynamic efficacy of the non-immobilized porphyrins towards T24 human BC cells was dependent on the wavelengths used. TMPyP and Zn-TMPyP showed higher efficiency under BL (417 nm, 2.5 J cm^−2^, IC_50_ values of 0.42 and 0.22 μm, respectively), while P1-C_5_ proved to be more active under RL (625 nm, 25 J cm^−2^, IC_50_ value of 0.14 μm). The observed photocytotoxicity is mainly related to the specific high production of singlet oxygen (rather than ROS), which is mainly responsible for cancer cell death through apoptosis.

The antiproliferative photodynamic activity of the porphyrins in the hybrid materials after irradiation with RL was similar or only slightly reduced when compared to the non-immobilized porphyrins. In general, porphyrin@GQDs demonstrated a higher PDT efficiency than porphyrin@GO hybrids, possibly due to their smaller size, which facilitates cell internalization. The most promising hybrid systems were Zn-TMPyP@GQDs, followed by P1-C_5_@GO and P1-C_5_@GQDs. Their low IC_50_ values prove that porphyrin functionalized carbon nanomaterials retain the high potential as PS for PDT, thus paving the way for future biomedical applications of such hybrids on cancer therapy, simplifying therapeutics formulation, increasing the stability of the compounds during distribution in the body after administration, and thus providing a greater chance to target the diseased tissue.

Results from the use of these carbon-based nanoplates suggest that these materials are useful for improving the stability of porphyrins in aqueous solutions. Moreover, these platforms present multiple functional groups that could be exploited for the conjugation of active-targeting molecules towards specific BC receptors (i.e., EGFR, HER-2, FGFR-3, etc.) [13]. A further advantage is their ability to absorb near-infrared light and convert it into heat, which is harmful to cells [48]. This phenomenon can be exploited to increase the antiproliferative activity of photosensitizers for their use in photodynamic-photothermal therapy, but further in-depth studies are needed.

## Figures and Tables

**Table 1:** Molar absorption coefficients of the three cationic porphyrin derivatives at their Soret band and at 417 nm (BL) and 625 nm (RL).

Porphyrin Derivatives	λ _Soret band (nm)_	ε (mol^−1^·dm^3^·cm^−1^)		
		λ _Soret band_	λ _Blue light_ (417 nm)	λ _Red light_ (625 nm)
TMPyP	423	98,823	90,688	1407
Zn-TMPyP	438	85,792	38,860	1395
P1-C_5_	465	44,468	11,955	5017

**Table 2:** Binding ratios of the porphyrin@GO and porphyrin@GQDs hybrids in PBS.

Binding Ratios	Porphyrin@Carbon Nanomaterial		
	TMPyP	Zn-TMPyP	P1-C_5_
Binding ratio of Porphyrin/GO	1/3.8	1/4.2	1/2
Binding ratio of Porphyrin/GQDs	2/1	3/1	2.6/1

## Data Availability

Not applicable.

## Supplementary Materials

The following are available online at https://www.mdpi.com/article/10.3390/pharmaceutics13091512/s1; Full characterization of porphyrins obtained at different synthetic steps; Figure S1: ^1^H NMR of Ni-P1 (300 MHz, CDCl_3_); Figure S2: ^13^C NMR of Ni-P1 (125 MHz, CDCl_3_); Figure S3: HRMS-ESI(+) of Ni-P1; Figure S4: ^1^H NMR of P1 (300 MHz, CDCl_3_); Figure S5: ^13^C NMR of P1 (125 MHz, CDCl_3_); Figure S6: HRMS-ESI(+) of P1; Figure S7: ^1^H NMR of P1-C_5_ (300 MHz, DMSO-d_6_); Figure S8: ^13^C NMR of P1-C_5_ (125 MHz, DMSO-d_6_); Figure S9: HRMS-ESI(+) of P1-C_5_; Figure S10: (a) Fluorescence spectra of GO at the studied conditions used for porphyrin@GO hybrids: here, 50 µL of GO (1 mg mL^−1^) were added to 1 mL of PBS solution and excited at two maxima of TMPyP (λ_max_ 423 nm) and Zn-TMPyP (λ_max_ 438 nm). (b) Comparison of fluorescence of GQDs and GO at the same concentration under a 366 nm lamp; Figure S11: (a) High magnification AFM image of GO flakes deposited onto the Si substrate. The black line and the colored arrows represent the cross-section and the measurement points used to calculate the thickness, respectively. (b) AFM height profile of the cross-section analysis. STEM images (transmission mode) of (c) GO and (d) GQDs. The red dotted line represents the circle circumscribing the coalesced GQDs in the microscopy analysis; Figure S12: Raman spectra (532 nm excitation) of the sheet-edge areas of TMPyP@GO hybrid; Figure S13: UV-Vis absorbance spectrum of a GQDs aqueous solution (0.1 µg mL^−1^). The inset highlights that this solution is transparent under daylight; Figure S14: Fluorescence spectra of an aqueous solution of GQDs (0.1 µg mL^−1^), excited at 330 nm at each 10 min (during 100 min). At these conditions, a 26% fluorescence quenching was observed; Figure S15: (a) Raman spectra (532 nm excitation) of free GQDs (blue line), Zn-TMPyP (green line), and Zn-TMPyP@GQDs (red line). (b) Corresponding data for P1-C_5_@GQDs; Figure S16: UV-Vis spectra of P1-C_5_ under increasing light irradiation doses of (a) RL (0–35 J cm^−2^) and (b) BL (0–20 J cm^−2^). BL had an irradiance of 17 mW cm^−2^ and RL had an irradiation of 12 mW cm^−2^; Figure S17: Antiproliferative photodynamic activity (25.0 J cm^−2^ of RL) of pre-irradiated P1-C_5_ at different concentrations; Figure S18: (a) Singlet oxygen production of non-immobilized porphyrins at concentrations 0.1 μm, 1.0 μm, and 5.0 μm under BL. (b) ROS production by the non-immobilized porphyrins tested at their IC_50_ under BL. Data are expressed as mean ± SD of at least two independent experiments carried out in triplicate. (* *p* < 0.0332, ** *p* < 0.0021, *** *p* < 0.0002, **** *p* < 0.0001; two-way ANOVA); Figure S19: Photo-proliferative activity of GQDs at different concentrations under RL.
