# Supplementary material for: Graphene Oxide and Graphene Quantum Dots as Delivery Systems of Cationic Porphyrins: Photo-Antiproliferative Activity Evaluation towards T24 Human Bladder Cancer Cells
Source: Pharmaceutics. 2021 Sep 18;13(9):1512. doi: 10.3390/pharmaceutics13091512 (PMC8470602; doi:10.3390/pharmaceutics13091512)
Supplement: Supplementary file 1 [file pharmaceutics-13-01512-s001.zip › pharmaceutics-1325648-supplementary.pdf]

pharmaceutics

Supplementary Materials: Graphene Oxide and Graphene Quantum Dots as Delivery Systems of Cationic Porphyrins: Photo-Antiproliferative Activ-ity Evaluation towards T24 Human Bladder Cancer Cells

Luca Menilli, Ana R. Monteiro, Silvia Lazzarotto , Filipe M. P. Morais, Ana T. P. C. Gomes, Nuno M. M. Moura, Sara Fateixa, Maria A. F. Faustino, Maria G. P. M. S. Neves, Tito Trindade and Giorgia Miolo

1. Characterization of Porphyrins: ^{1}H NMR, ^{13}C NMR, HRMS-ESI(+)

Full characterization of porphyrins obtained at different synthetic steps (see Materials and Methods, Section 2.2)

- **TPyP - Yield:** 11%. **^{1}H-NMR** ($CDCl_3/CD_3OD$, 300 MHz): *δ* ppm = 9.03 (d, 8H, *J* = 4.6 Hz, H-*m*-Py), 8.89 (s, 8H, $H_β$), 8.23 (d, 8H, *J* = 4.6 Hz, H-*o*-Py), -2.93 (s, 2H, *N*H). **ESI-MS(+):** *m/z* 619.3 $[M+H]^+$.

- **TMPyP - Yield:** 70%. **^{1}H NMR** (DMSO-d$_6$,, 300 MHz): *δ* (ppm) = 9.49 (d, 8H, *J* = 6.7 Hz, H-*o*-Py), 9.20 (s, 8H, $H_β$), 9.00 (d, 8H, *J* = 6.7 Hz, H-*m*-Py), 4.73 (s, 12H, CH_3), -3.11 (s, 2H, *N*H). **ESI-MS(+):** *m/z* = 169.6 $[M]^{4+}$.

- **Zn-TMPyP - Yield:** 90%. **^{1}H NMR** (DMSO-d$_6$, 300 MHz): *δ* (ppm) = 9.43 (d, 8H, *J* = 6.7 Hz, H-*o*-Py), 9.10 (s, 8H, $H_β$), 8.92 (d, 8H, *J* = 6.7 Hz, H-*m*-Py), 4.72 (s, 12H, CH_3). **ESI-MS(+):** *m/z* 185.1 $[M]^{4+}$.

- **β-Br$_4$TPP - Yield:** 54%. **^{1}H-NMR** ($CDCl_3$, 300 MHz): *δ* (ppm) = 8.70 (d, 4H, *J* = 1.3 Hz, $H_β$), 8.20-8.16 (m, 8H, H-*o*-Ph), 7.81-7.75 (m, 12H, H-*m,p*-Ph), -2.82 (s, 2H, *N*H). **ESI-MS(+):** *m/z* 931.1 $[M+H]^+$.

- **Ni-β-Br$_4$TPP - Yield:** 67%. **ESI-MS(+):** *m/z* 987.1 $[M]^+$.

- **Ni-P1 - Yield:** 48%. **^{1}H-NMR** ($CDCl_3$, 300 MHz): *δ* (ppm) = 8.76 (s, 4H, $H_β$), 8.47 (dd, 8H, *J* = 1.5 and 3.0 Hz, H-*o*-Py), 7.99-7.96 (m, 8H, H-*o*-Ph), 7.77-7.75 (m, 12H, H-*m,p*-Ph), 7.11 (s, 4H, Ha), 6.95 (dd, 8H, *J* = 1.5 and 3.0 Hz, H-*m*-Py) (Figure S1). **^{13}C NMR** ($CDCl_3$, 125 MHz): *δ* (ppm) = 149.5, 148.8, 142.1, 140.3, 139.4, 136.4, 134.4, 132.7, 131.8, 128.8, 126.2, 124.9, 116.9 (Figure S2). **HRMS-ESI(+):** *m/z* calcd for $C_{72}H_{45}N_8Ni$: 1079.3121 $[M+H]^+$; 1079.3091 found (Figure S3).

- **P1 - Yield:** 52%. **^{1}H-NMR** ($CDCl_3$, 300 MHz): *δ* (ppm) = 8.85 (s, 4H, $H_β$), 8.50 (d, 8H, *J* = 6.0 Hz, H-*o*-Py), 8.24-8.22 (m, 8H, H-*o*-Ph), 7.87-7.81 (m, 12H, H-*m,p*-Ph), 7.14 (s, 4H, Ha), 6.97 (d, 8H, *J* = 6.0 Hz, H-*m*-Py), -2.58 (s, 2H, *N*H) (Figure S4). **^{13}C NMR** ($CDCl_3$, 125 MHz): *δ* (ppm) = 149.6, 142.2, 141.8, 138.9, 135.5, 135.4, 135.2, 134.2, 133.9, 133.8, 133.7, 133.6, 133.0, 132.7, 128.8, 128.1, 127.7, 127.5, 127.7, 127.5, 127.43, 127.39, 127.27, 124.9, 122.9, 119.6, 118.6 (Figure S5). **HRMS-ESI(+):** *m/z* calcd for $C_{72}H_{47}N_8$: 1023.3924 $[M+H]^+$; 1023.3891 found (Figure S6).

- **P1-C$_5$ - Yield:** 74%. **^{1}H-NMR** (DMSO-d$_6$, 300 MHz)**:** *δ* ppm = 9.12 (d, 8H, *J* = 6.0 Hz, H-*o*-Py), 9.02 (s, 4H, $H_β$), 8.31 (d, 8H, *J* = 6.0 Hz, H-*o*-Ph), 7.97-7.95 (m, 12H, H-*m,p*-Ph), 7.77 (d, 8H, *J* = 6.0 Hz, H-*m*-Py), 7.02 (s, 4H, Ha), 4.67 (t, 8H, *J* = 6.0 Hz, $C_α\underline{H_2}$), 2.00 (quintet, 8H, *J* = 9.0 Hz, $C_β\underline{H_2}$), 1.45-1.30 (m, 16H, $C_{γ,δ}\underline{H_2}$), 0.97 (t, 2H, *J* = 6.0 Hz, $C\underline{H_3}$), -2.65 (s, 2H, *N*H) (Figure S7). **^{13}C NMR** (DMSO-d$_6$, 125 MHz): *δ* (ppm) = 155.7, 148.5, 145.0, 142.5, 141.5, 139.0, 133.9, 133.0, 129. 7, 129.6, 129.2, 128.9, 128.2, 120.1, 119.6, 117.2, 60.8, 34.8, 30.8, 29.5, 28.1, 22.1, 14.4 (Figure S8). **HRMS-ESI(+):** *m/z* calcd for for $C_{92}H_{90}N_8$: 326.6817 $[M]^{4+}$; 326.6817 found (Figure S9).

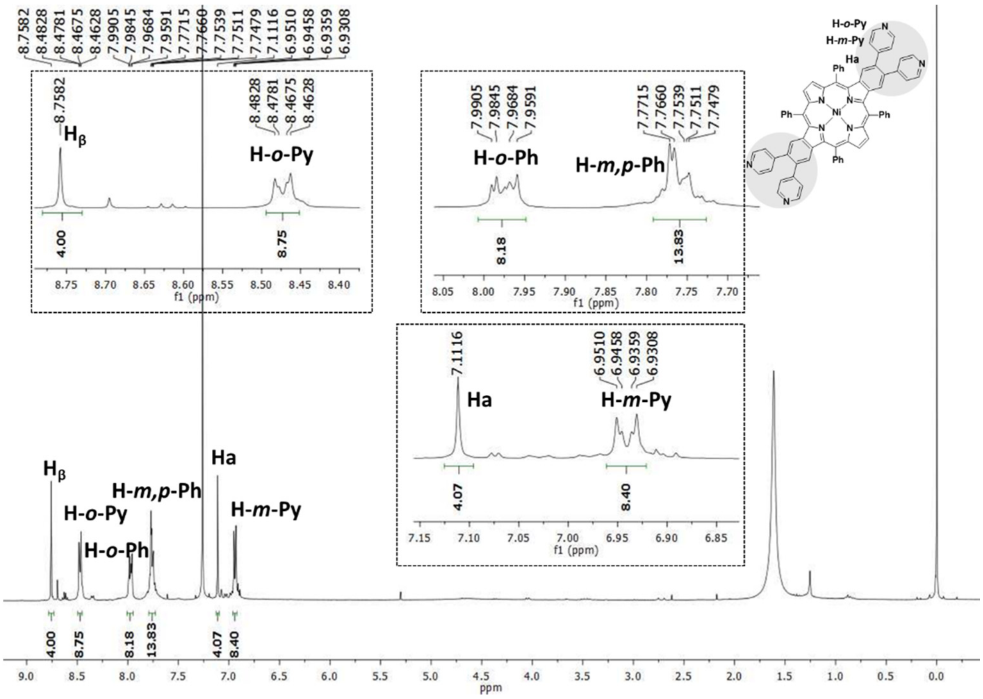

Figure S1. ^{1}H NMR of Ni-P1 (300 MHz, $CDCl_3$).

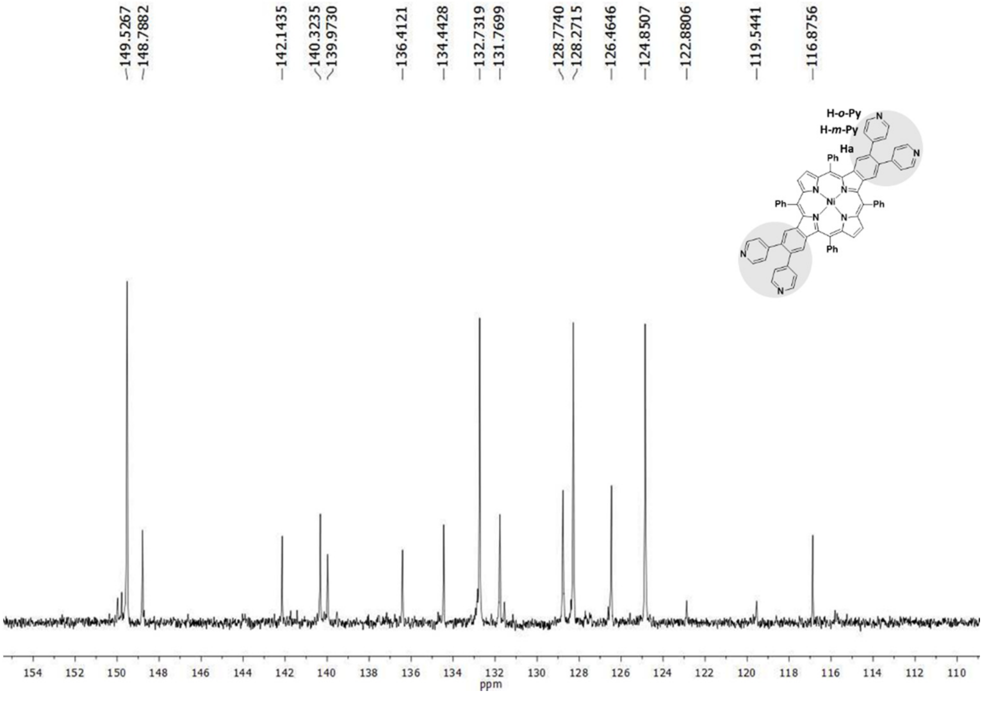

Figure S2. ^{13}C NMR of Ni-P1 (125 MHz, $CDCl_3$).

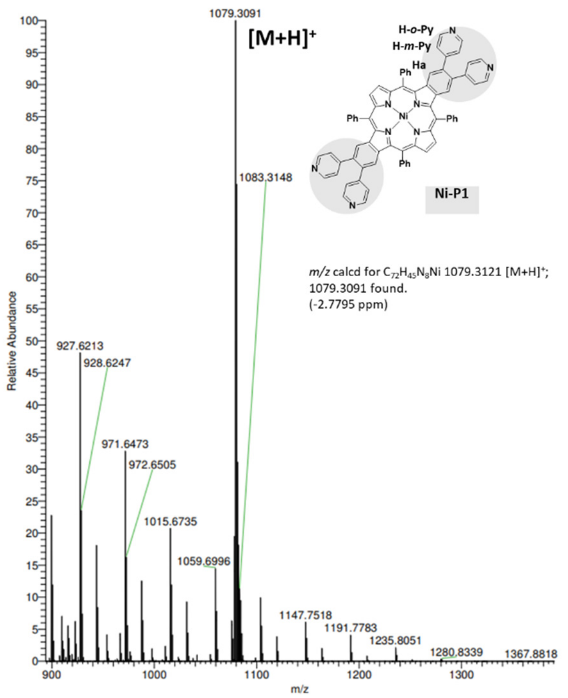

Figure S3. HRMS-ESI(+) of Ni-P1.

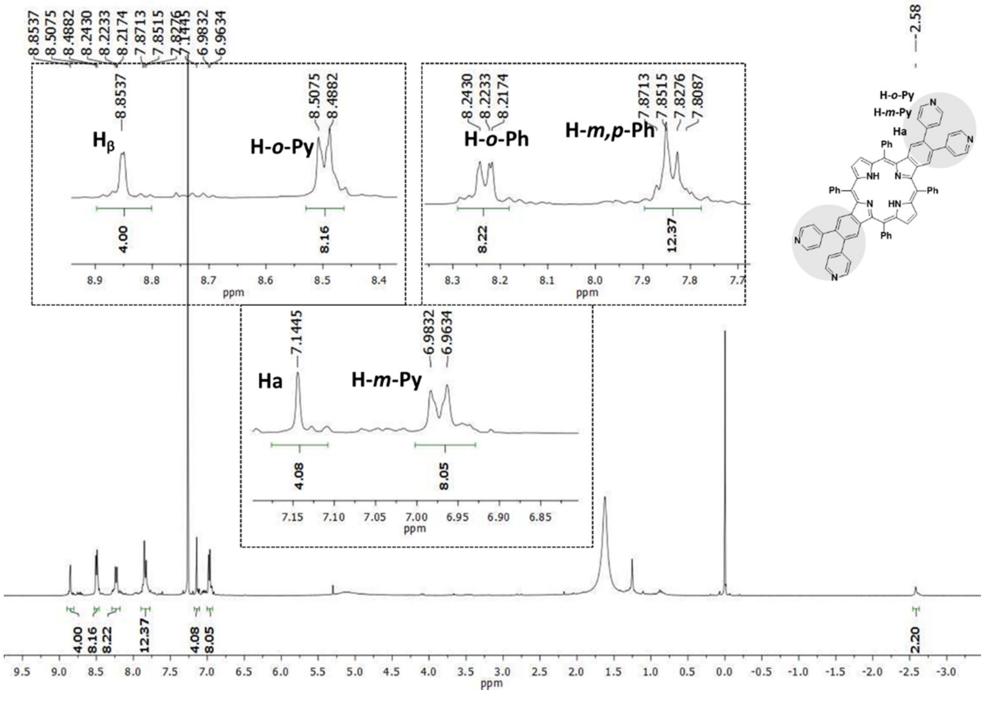

Figure S4. ^{1}H NMR of P1 (300 MHz, $CDCl_3$).

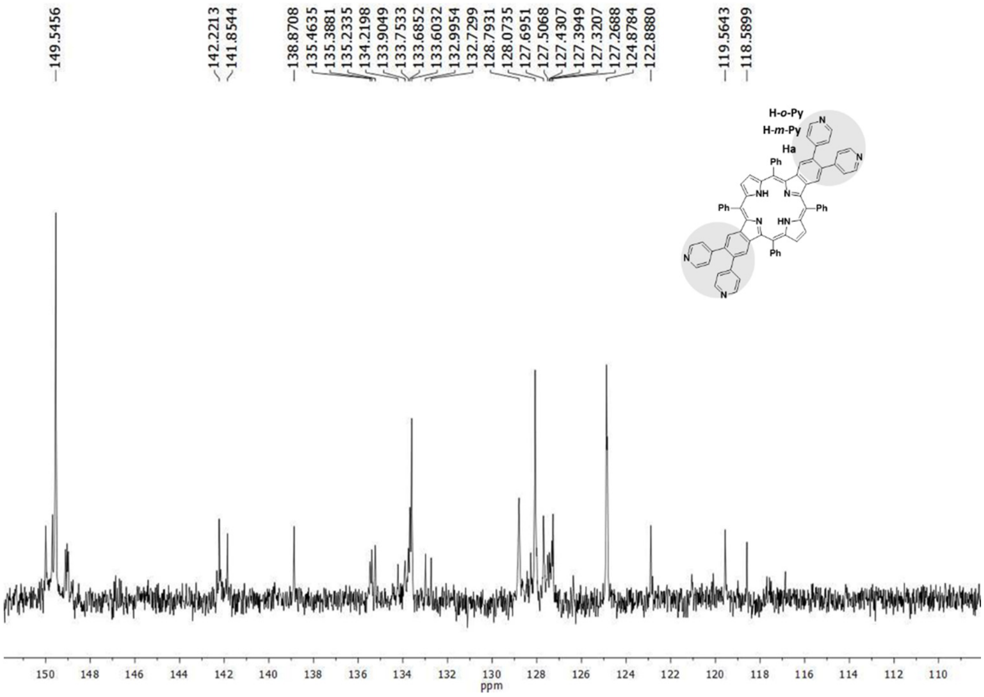

Figure S5. ^{13}C NMR of P1 (125 MHz, $CDCl_3$).

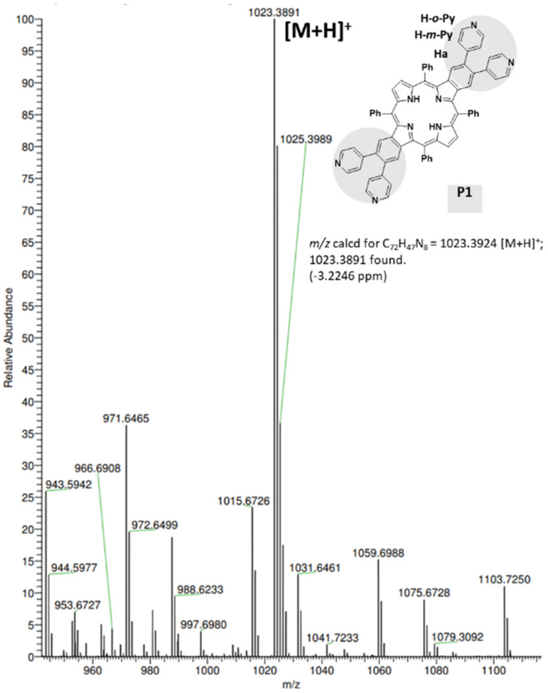

Figure S6. HRMS-ESI(+) of P1.

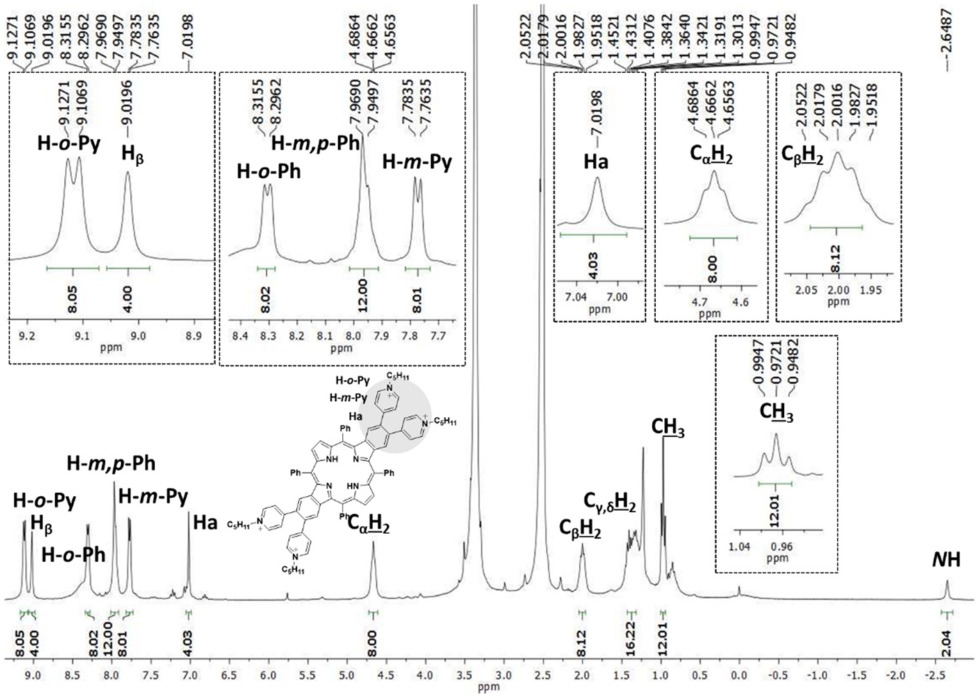

Figure S7. ^{1}H NMR of P1-C_5 (300 MHz, DMSO-d_6).

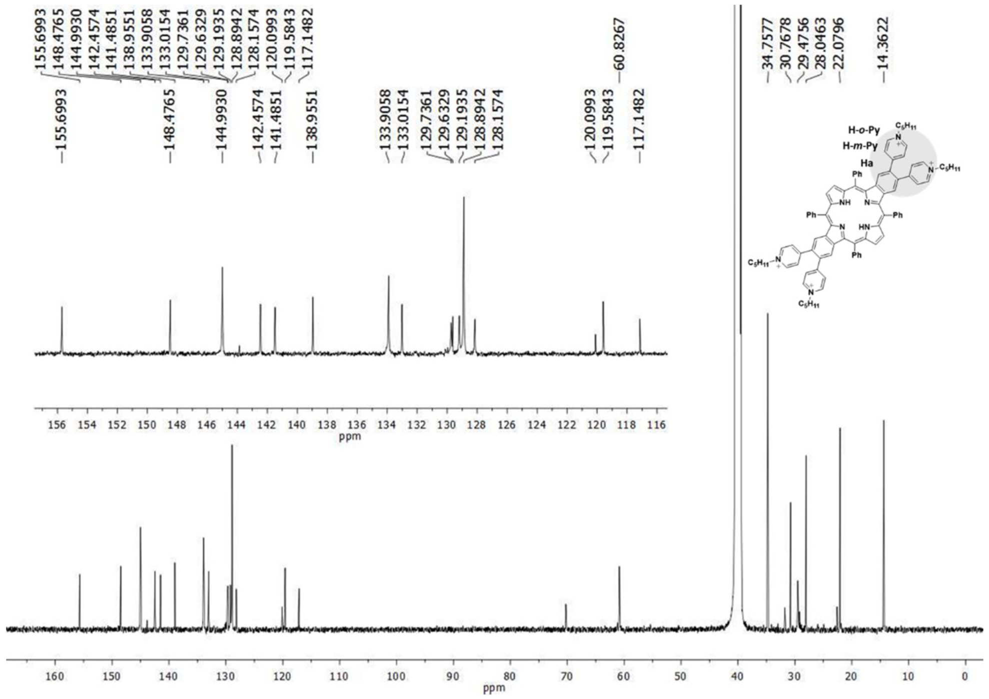

Figure S8. ^{13}C NMR of P1-C_5 (125 MHz, DMSO-d_6).

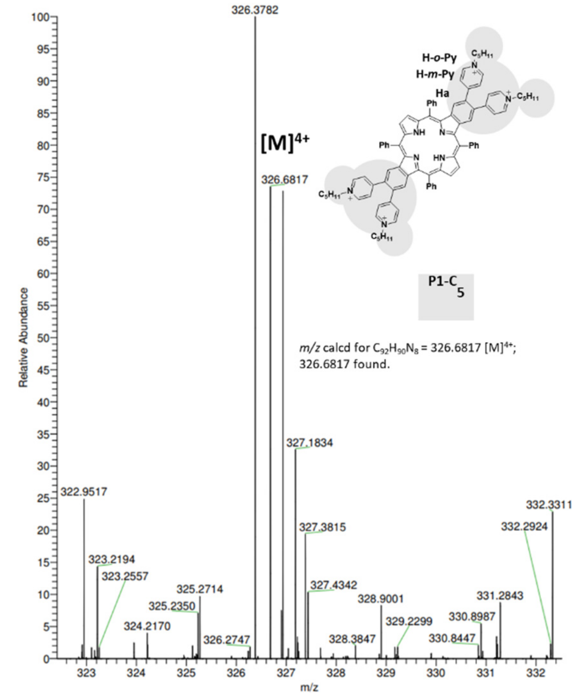

Figure S9. HRMS-ESI(+) of P1-C_5.

2. Characterization of Neat GO/GQDs and of Hybrid Nanomaterials

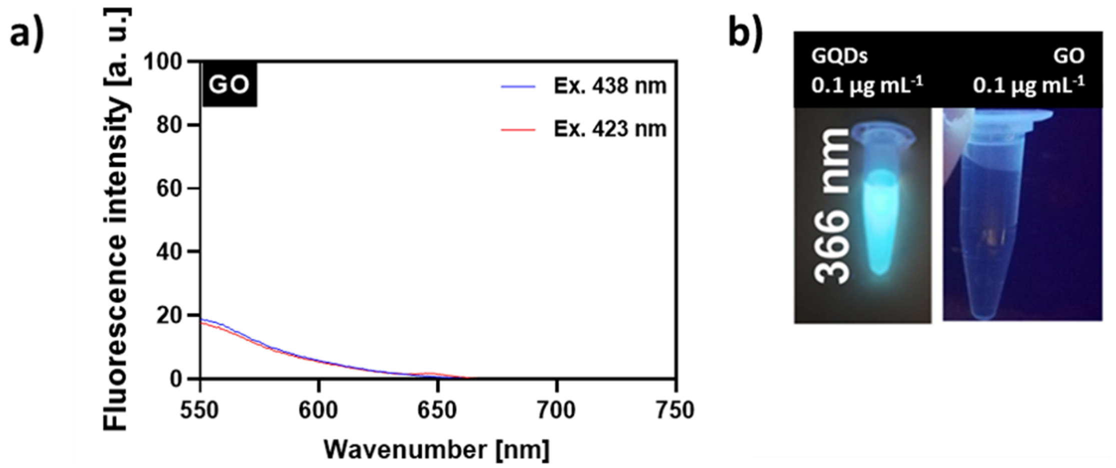

Figure S10. a) Fluorescence spectra of GO at the studied conditions used for porphyrin@GO hybrids: here, 50 µL of a GO (1 mg mL^{-1}) were added to 1 mL of PBS solution and excited at two maxima of TMPyP (λ_{max} 423 nm) and Zn-TMPyP (λ_{max} 438 nm). **b**) Comparison of fluorescence of GQDs and GO at the same concentration under a 366 nm lamp.

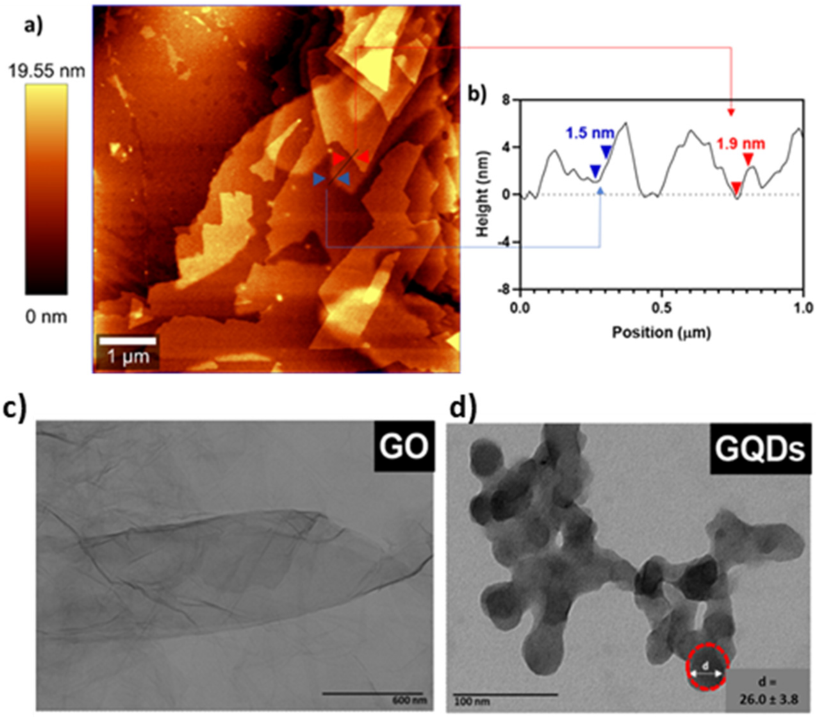

Figure S11. **a**) High magnification AFM image of GO flakes deposited onto the Si substrate. The black line and the colored arrows represent the cross-section and the measurement points used to calculate the thickness, respectively. **b**) AFM height profile of the cross-section analysis. STEM images (transmission mode) of **c**) GO and **d**) GQDs. The red dotted line represents the circle circumscribing the coalesced GQDs in the microscopy analysis.

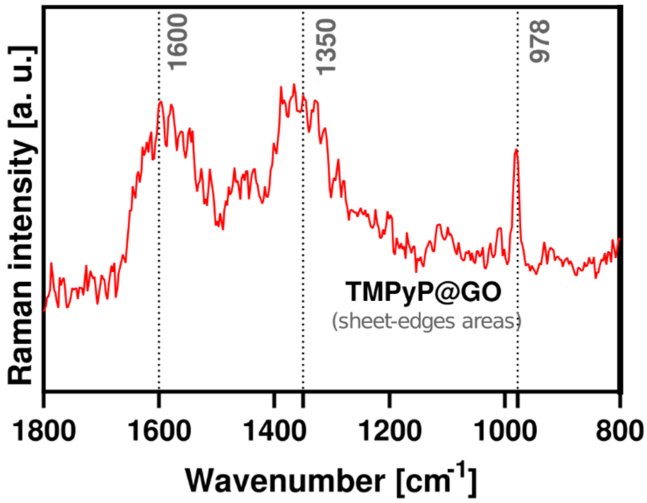

Figure S12. Raman spectra (532 nm excitation) of the sheet-edge areas of TMPyP@GO hybrid.

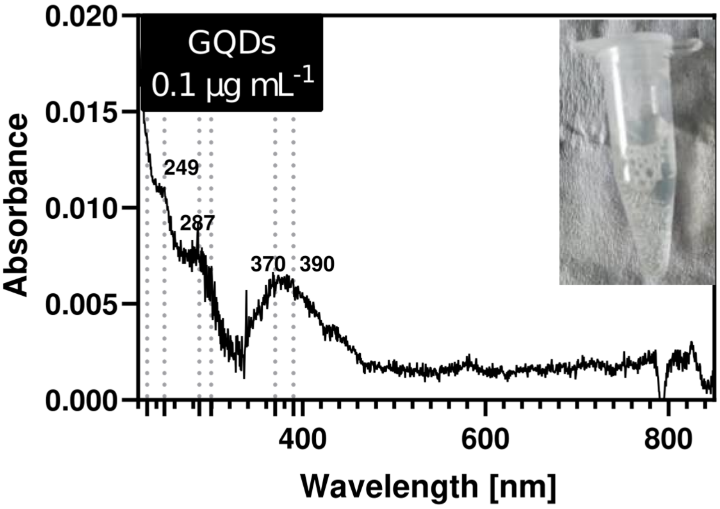

Figure S13. UV-Vis absorbance spectrum of a GQDs aqueous solution (0.1 µg mL^{-1}). The inset highlights that this solution is transparent under daylight.

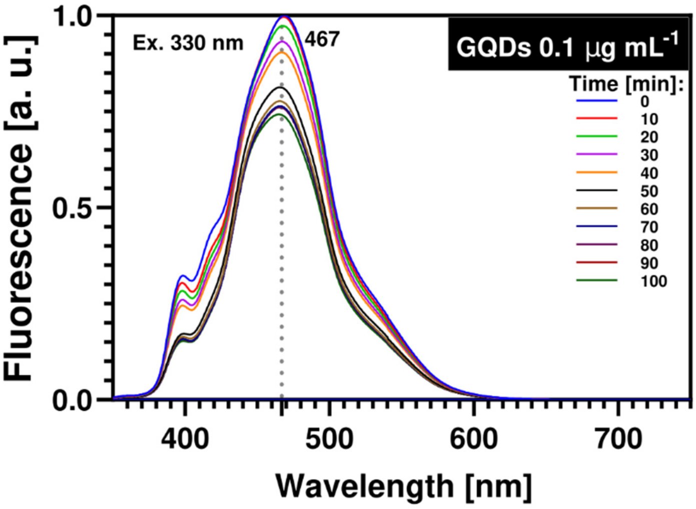

Figure S14. Fluorescence spectra of an aqueous solution of GQDs (0.1 µg mL^{-1}), excited at 330 nm at each 10 min (during 100 min). At these conditions, a 26% of fluorescence quenching was observed.

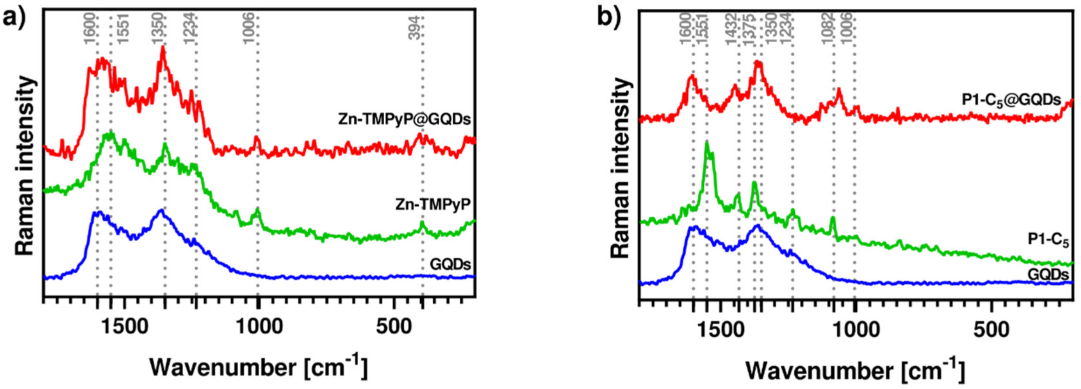

Figure S15. a) Raman spectra (532 nm excitation) of free GQDs (blue line), Zn-TMPyP (green line) and Zn-TMPyP@GQDs (red line). **b**) Corresponding data for P1-C_5@GQDs.

3. Photocytotoxicity Biological Assays

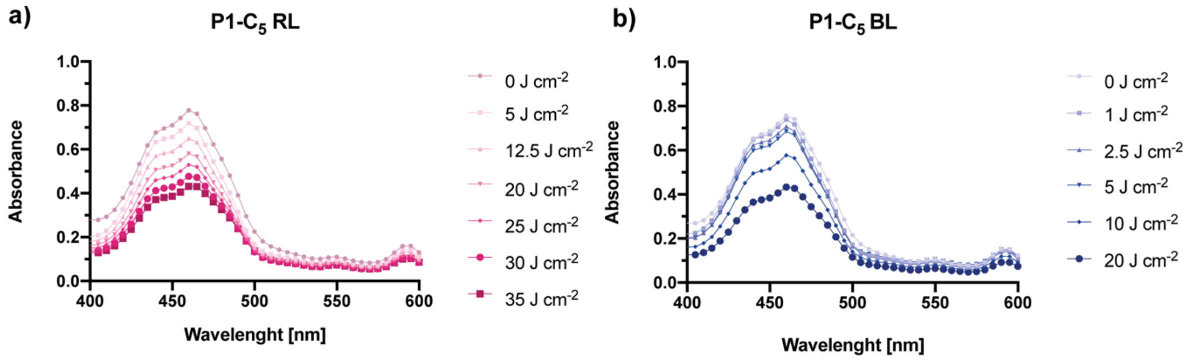

Figure S16. UV-Vis spectra of P1-C_5 under increasing light irradiation doses of a) RL (0–35 J cm^{-2}) and b) BL (0–20 J cm^{-2}). BL had an irradiance of 17 mW cm^{-2} and RL had an irradiation 12 mW cm^{-2}.

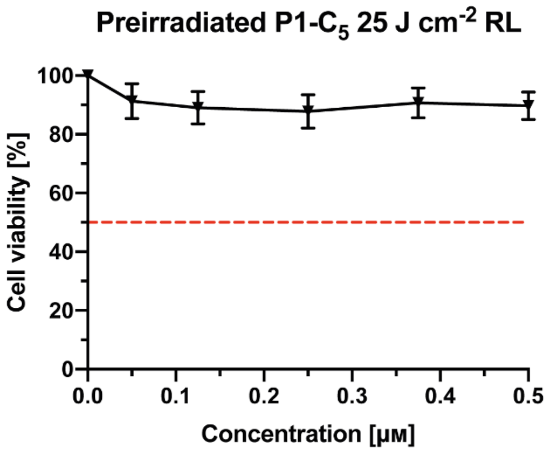

Figure S17. Antiproliferative photodynamic activity (25.0 J cm^{-2} of RL) of pre-irradiated P1-C_5 at different concentrations.

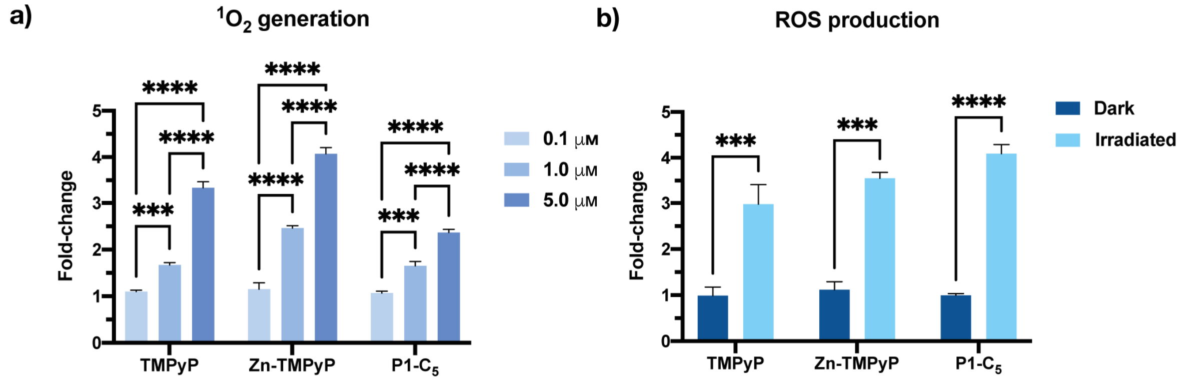

Figure S18. a) Singlet oxygen production of non-immobilized porphyrins at concentrations 0.1 µM, 1.0 µM, 5.0 µM under BL. b) ROS production by the non-immobilized porphyrins tested at their IC_{50} under BL. Data are expressed as mean ± SD of at least two independent experiments carried out in triplicate. (* $p < 0.0332$, ** $p < 0.0021$, *** $p < 0.0002$, **** $p < 0.0001$; two-way ANOVA).

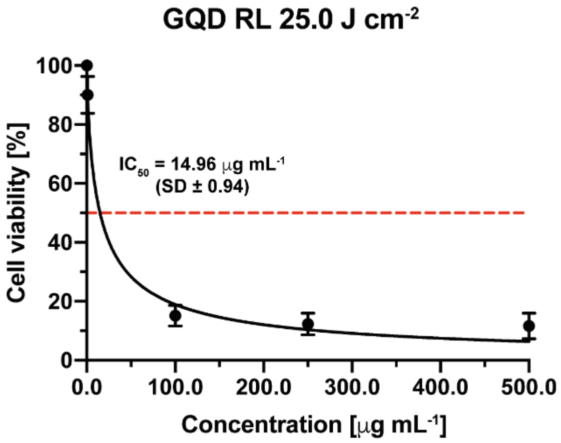

Figure S19. Antiproliferative activity of GQDs at different concentrations under RL.
